# Interface Nucleus Templating of Modular Intermetallic Morphologies: Chemical Pressure Complementarity, Columnar Domains, and Complex Disorder in Y_13_Ag_42.7_Zn_29.7_

**DOI:** 10.1021/acs.inorgchem.5c03538

**Published:** 2025-10-25

**Authors:** Rie T. Fredrickson, Daniel C. Fredrickson

**Affiliations:** Department of Chemistry, 5228 University of Wisconsin-Madison, 1101 University Avenue, Madison, Wisconsin 53706, United States

## Abstract

One element of the diversity of intermetallic phases is the formation of complex structures from the assembly of fragments of simpler structures. Recently, we devised the Interface Nuclear Approach as a model for understanding such modular arrangements, in which the intergrowth of different structures is driven by chemical pressure (CP) relief at shared motifs at the domain interfaces, referred to as interface nuclei. In this Article, we present the synthesis, crystal structure, and CP analysis of a new compound that expands on this theme, Y_13_Ag_42.7_Zn_29.7_. Its hexagonal structure contains interpenetrating domains based on the CaPd_5+*x*_ and EuMg_5_ types. The CaPd_5+*x*_-based regions are reminiscent of the lamellar intergrowth structures previously observed in the Y–Ag–Zn system. In Y_13_Ag_42.7_Zn_29.7_, however, the domains have a different morphology, forming columns that adopt a hexagonal rod-packing. The geometrical features of the remaining spaces are assigned, using the program *GrowDomain*, to the cores of trigonal units of the EuMg_5_ type, while layers of disordered atoms occur at heights along *z* where the parent structures are mismatched. At the CaPd_5+*x*_-type/EuMg_5_-type interfaces, simple interface nucleus motifs with strong CP-complementarity can be identified, while their distribution within the parent structures supports the notion of templated architectures in modular intermetallics.

## Graphical abstract

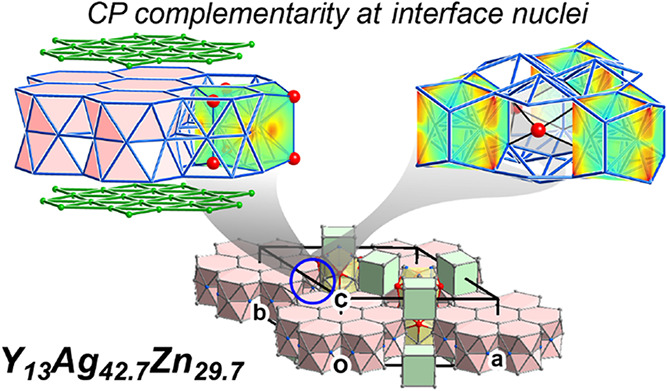

## 1 Introduction

In molecular chemistry, it is standard practice to build complex arrangements from simpler units drawing on well-defined rules for assembly. In materials chemistry, similarly, one frequently refers to building blocks of solid state structures, and in metal–organic frameworks, structural architectures can even be designed from the geometrical arrangements of docking sites on organic ligands and the preferred coordination environments of the metal centers.^1^ The chemist’s drive to thus arrange and connect molecular-scale units in new ways can, in fact, be extended to an unexpected realm: the chemistry of intermetallic phases. While elemental metals generally crystallize in simple sphere packings, their combination leads to a vast structural diversity.^2,3^ Included in this diversity are complex arrangements in which units of simpler intermetallic structures are combined,^4–9^ suggestive of a modular chemistry of intermetallics that could be applied to the design of new compounds. However, this prospect is limited by the need for an understanding of the chemical factors stabilizing complex structures over their separate parent structures and how they can be harnessed in synthetic endeavors.

Recently, we introduced the Interface Nucleus Approach as a framework for gaining this understanding of modular intermetallic structures.^10,11^ In this model, we consider how the intergrowth of two parent structures can be facilitated by their sharing of a geometrical motif (Figure 1). The presence of this unit at the edge of a domain of one parent provides an opportunity, at least conceptually, for the nucleation of a domain of the second parent structure with the shared unit serving as a coherent interface. We thus refer to these units as *interface nuclei.* ^12^ In this scheme, the search for driving forces for combining structures into larger-scale assemblies then begins with examination of the ways the atoms within the interface nuclei are influenced by the new context offered by the intergrowth.

**Figure 1:**
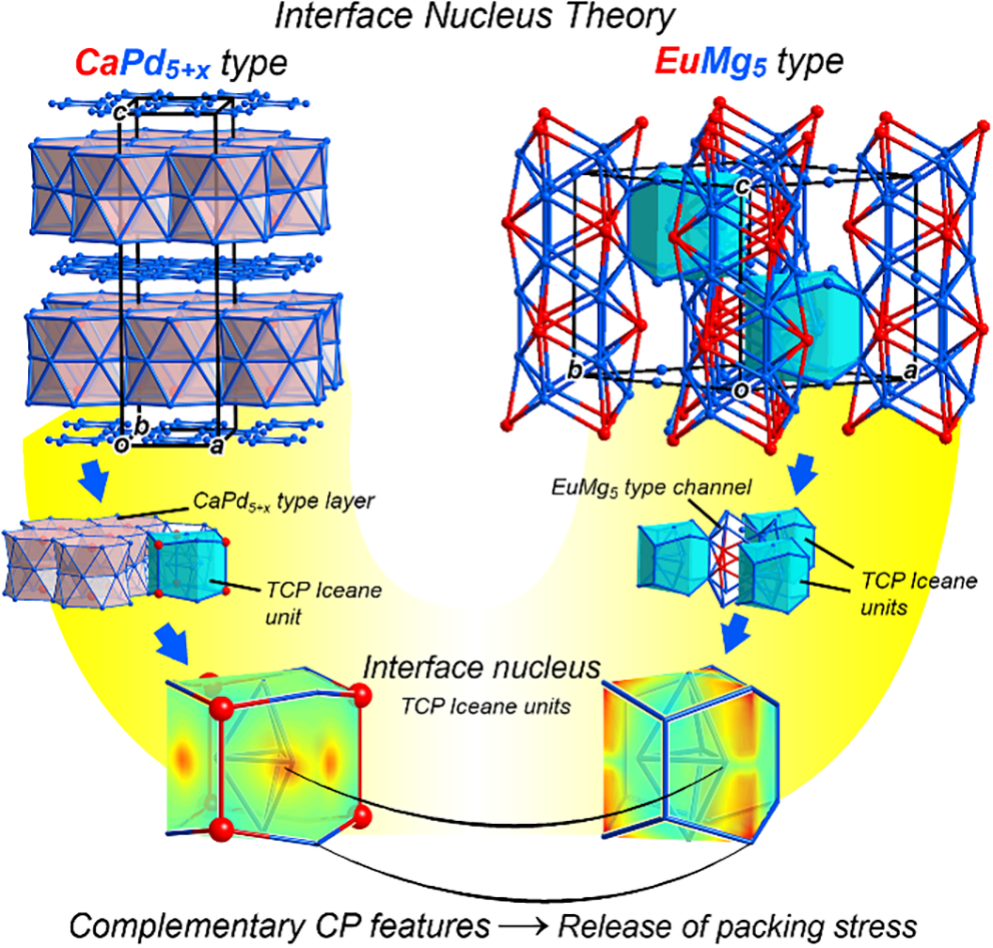
Interface nucleus approach, illustrated with the CaPd_5+*x*_- and EuMg_5_-types as parent structures. The structures share a common unit, a tetrahedrally close packed (TCP) motif bounded by an iceane-like cage, which can serve as nucleation points for interfaces between the structures. Such interfaces offer opportunities for the relief of complementary chemical pressure features that the unit experiences in the two structures separately.

Through a series of examples, we have seen that atomic packing issues within the parents provide an impetus for merging structures at such interface nuclei. Here, the DFT-Chemical Pressure (CP) method^13^ is used to construct maps of the local pressures within crystal structures and interpret them in terms of interatomic pressures arising from atomic packing constraints, essentially resolving the internal pressure of a material into a competition between overly short and overly stretched contacts between atoms. DFT-CP analyses of the formation of the σ-phase structure from the Cr_3_Si and Al_3_Zr_4_ types,^14^ YNi_3_ and similar layered intergrowths of the CaCu_5_ and Laves phase types,^15^ the Ca_6_Cu_6_Al_5_ type from the MgZn_2_ and Mg_2_Zn_11_ types,^16,17^ and other cases, reveal points of complementarity in the CP features of the interface nuclei in the parent structures.^11^ The opportunity for relief of atomic packing tensions through the merging of the two structures at the interface nucleus motivates the formation of the modular structure, a principle that can be applied to the discovery of new modular phases.^10^ Meanwhile, the details of that complementarity appear to guide the orientation of the domain interfaces, while the distribution of the interface nuclei in the parent structures templates the domain morphology.

The utility of this approach for materials design, though, depends largely on the degree that the domain morphologies of modular intermetallic structures can be influenced by synthetic parameters, particularly the composition of the sample. In this Article, we present an illustration of this structural tunability in the Y–Ag–Zn system with the synthesis, structure determination, and CP analysis of Y_13_Ag_42.7_Zn_29.7_.^18^ Its structure combines an ordered framework similar to the Ta_13_Co_40_Si_31_ type^19–23^ with layers of significant positional disorder. Like the three earlier compounds we described in this system, YAg_2.79_Zn_2.80_, YAg_2.44_Zn_3.17_, and YAg_2.71_Zn_2.71_, the structure contains domains based on the CaPd_5+*x*_ type^24^ linked by units from the Mg_2_Zn_11_ type.^25^ However, while in all of the earlier cases the domains are lamellar, the larger Ag content of Y_13_Ag_42.7_Zn_29.7_ results in a hexagonal rod-packing morphology.

To track down the origins of this structural change, we will introduce a new program for analyzing modular structures in terms of blocks of their parent structures, *GrowDomain*. With this approach, the hexagonal pattern of columns will be traced to the templating effect of triangular domains of a third structure type, the EuMg_5_ type (Figure 1), that are absent in the earlier lamellar structures with lower Ag content (see the Supporting Information for a comparison of the positions of these compounds within the compositional space of the Y–Ag–Zn system). These domains meet edgewise with the CaPd_5+*x*_-type regions at interface nuclei with strong CP complementarity. We will also see that this combination of the CaPd_5+*x*_ and EuMg_5_ types provides an explanation for the disordered atomic layers in terms of points of mismatch between the structures. In these ways, Y_13_Ag_42.7_Zn_29.7_ affirms aspects of the Interface Nucleus Approach for modular intermetallics while demonstrating how such structures can evolve under elemental substitution.

## 2 Experimental Section

### 2.1 Synthesis

In the synthesis of Y_13_Ag_42.7_Zn_29.7_, Y (Strem chemicals, 99.9%), Ag (Aldrich, 99.99%) and Zn (Alfa Aesar, 99.9%) were weighed out in the molar ratio of Y:Ag:Zn = 13:38.6:27.2 in an Ar-filled glovebox (total mass ∼0.5 g). The materials were pressed into pellets and placed into fused silica tubes under Ar, which were then evacuated and sealed. The samples were annealed at 400 °C for 24 h for initiating reactions among the elements, heated up to 600 °C for 192 h, then finally cooled at a rate of 50 °C/hour to ambient temperature. A synthesis starting with a molar ratio closer to the compound’s stoichiometries was also attempted, but required higher temperatures (800 °C for 24 h, then 1000 °C for 168 h, and finally slow cooling to ambient temperature), likely due to the higher Ag content.

### 2.2 Powder X-ray Diffraction Analysis

The phase purity of the samples was assessed using powder X-ray diffraction analysis. Each sample was ground to a fine powder and placed on a zero-background plate. Diffraction intensities were measured on a Bruker D8 Advance Powder Diffractometer fitted with an LYNXEYE detector, using Cu Kα radiation (λ = 1.5418 Å) at ambient temperature. The exposure time of 1.0 s per 0.010° increment was used over the 2θ range of 20–80°.

### 2.3 Wavelength Dispersive X-ray Spectroscopy

To determine the elemental composition of the title compound, wavelength dispersive X-ray spectroscopy (WDS) was performed on a sample revealed by powder X-ray diffraction pattern to contain it as the major phase. In preparation for the WDS measurements, a small amount of material was suspended in a conductive epoxy at one end of a short segment of aluminum tubing. Once the epoxy had hardened, the sample was ground down to produce a flat surface and polished using a diamond lapping film (Precision Surface International Inc., 0.5 μm), and finally carbon coated. WDS measurements guided by back scattered electron images were taken with a Cameca SX-Five FE-EPMA Microprobe. Elemental Y, Ag, Zn, and Si were used as standards for Y Lα, Ag Lα, Zn Kα, and Si Kα transitions, respectively.

### 2.4 Single Crystal X-ray Diffraction Analysis

Single crystal X-ray diffraction data for crystals selected from the reaction products were collected on a Bruker Quazar SMART APEX2 diffractometer using Mo Kα radiation (λ = 0.71073 Å) at ambient temperature. The collection and integration of the data set were performed using the APEX2 Ver. 2014.11–033 and SAINT Ver. 8.34A34 software, and the absorption correction was carried out using SADABS Ver.2014/535. The structures were solved with the charge flipping algorithm^26,27^ using the program SUPERFLIP^28^ and refined on F^2^ with JANA2006.^29^ Further details regarding the refinements and crystallographic information are given in Table 1 and the Supporting Information. To explore the role of vibrations in the structure’s disorder, a data set on the same crystal was collected at *T* = 100 K on a Bruker D8 VENTURE Photon III four-circle diffractometer, using a Diamond IμS Mo Kα microfocus source.

**Table 1:** Crystallographic Data for Y_13_Ag_42.7_Zn_29.7_

chemical formula	Y_13_Ag_42.7(4)_Zn_29.7(4)_
WDS composition	Y_15(1)_Ag_50.1(3)_Zn_34.8(2)_
*a* (Å)	19.760(7)
*c* (Å)	9.079(3)
volume (basic cell, Å^3^)	3070.1(19)
*Z*	2
space group	*P*6/*mmm*
crystal dimensions (mm^3^)	0.122 × 0.100 × 0.094
crystal color, habit	silver, prismatic
data collection temperature	ambient
radiation source, λ (Å)	Mo Kα microfocus, 0.71073
absorption correction	numerical
min/max transmission	0.0860/0.2045
θ_min_, θ_max_	2.5396/30.1086
refinement method	*F* ^2^
number of reflections	113296
unique refl. [*I* > 3σ(I), all]	9542
*R*_int_ [I > 3σ(I), all]	3.62, 3.65
number of parameters, constraints	139, 40
*R*[I > 3σ(I)], *R*_w_[I > 3σ(I)]	1.66, 1.89
*R*(all), *R*_w_(all)	5.05, 5.09
*S*[I > 3σ(I)], *S*(all)	1.93, 1.98
Δρ_max_, Δρ_min_ (electrons/Å^3^)	1.36, −1.17

### 2.5 DFT-Chemical Pressure (CP) Analysis

DFT-CP analysis was performed for YAg_5_ in a simplified version of the CaPd_5+*x*_ type^24^ and the EuMg_5_ type^30^ from LDA-DFT electronic structures calculated with the ABINIT package.^31–33^ In each case, crystal structures were first geometrically optimized beginning with the relaxation of the atomic positions with fixed cell parameters and then releasing all structural parameters. Next, single point calculations were carried out on the optimized structures as well as slightly contracted and expanded versions (linear scales 0.995 and 1.005) to obtain the ground state electron densities, Kohn–Sham potential components, and wave functions used as input by the CPpackage program. Hartwigsen-Goedecker-Hutter norm-conserving pseudopotentials^34^ and the Teter LDA functional^35^ (the standard platform for CP calculations) were employed. The energy cutoff was set to 60 Ha, while Γ-centered 9 × 9 × 5 and 5 × 5 × 5 meshes were used for the CaPd_5+*x*_-based and EuMg_5_-type structures, respectively.

DFT-CP maps were generated from the ABINIT output with the CPpackage3 program,^36^ employing the core-unwarping procedure,^37^ mapping of nonlocal energy components,^38^ and self-consistent treatment for the spatial mapping of the *E*_Ewald_+*E*_α_ contributions.^13^ Interatomic pressures were determined by averaging the pressure maps within contact volumes constructed using the iterative binary Hirshfeld procedure,^13^ with the necessary free ion calculations being performed with the Ab initio Pseudopotentials Engine (APE).^39^ The averaged map was then projected onto atomic-centered spherical harmonics for visualization of the CP distribution around each atom as a radial plot (*l*_max_ = 4). The *CP*_*interface*_ function, representing the sums of the CP features directed through a specified surface, was calculated for the convex hulls of geometrical units following the procedure described in ref 40.

### 2.6 Analysis of the Domain Structure

The atomic positions of Y_13_Ag_42.7_Zn_29.7_ were mapped to counterparts in the CaPd_5+*x*_- and EuMg_5_-type parent structures using the *GrowDomain* program.^41^ The process begins by identifying a shared geometrical motif in the complex structure and a parent structure. The ToposPro program^42^ is then used to generate graph-representations of the motif in the two structures as well as the atomic positions of the motif together with several shells of atomic neighbors (creating clusters of >1000 atoms). *GrowDomain* uses these graphs and atomic coordinates to determine an isomorphic mapping between atoms of the initially identified shared motif.

The program then begins an iterative process to build a progressively larger shared domain between the structures. First, a transformation matrix is refined for mapping the atomic coordinates of the corresponding atoms in one structure to those in the other structure (with the origins shifted to the centers of the shared domains). Once this transformation is determined, the structures are placed in a common coordinate system, and the program explores the atomic positions in the two structures within a set distance, *r_search*, of atoms in the shared domain. Pairs of these atoms are added to the list of those mapped between the two structures when, upon superimposing the structures, they lie within (1) a chosen cutoff distance, *d_max*, and (2) a set number, *n*, of times the average mismatch distance between the atoms currently in the shared domain.^43^ After all such atoms are identified, the list of atoms in the shared domains and their mappings is updated, and the process cycles back to the refinement of the transformation matrix and a new iteration begins. The process is completed when the next cycle does not identify any new atoms.

In the case of the Y_13_Ag_42.7_Zn_29.7_, the structure was first reduced to its basic framework through the removal of all atoms exhibiting positional disorder. A tricapped trigonal prism from a EuMg_5_-type column was then selected as a potential seed for a larger EuMg_5_-type domain and a counterpart in this parent type was identified. Meanwhile, a double-hexagonal antiprism from the center of a CaPd_5+*x*_-based column was extracted as a seed for the investigation of the full extent of the CaPd_5+*x*_-based domains. For the *GrowDomain* iterations for the EuMg_5_-type domain, *r_search*, *d_max*, and *n* were set to 4.0 Å, 0.8 Å, and 3, respectively. The corresponding values for the CaPd_5+*x*_-based domain were 4.0 Å, 0.8 Å, and 5 (with the larger value of *n* adjusting for a closer match between the structures in the early iterations).

## 3 Results and Discussion

### 3.1 Synthetic Results

In our earlier studies in the Ag- and Zn- rich side of the Y–Ag–Zn system,^18^ we encountered several new modular structures, while hints of several others were apparent in the powder patterns and metallographic analysis. The prospect of an even broader modular chemistry inspired us to synthetically explore the other structures in this system. The reaction products of these new syntheses were air stable ingots with a silver, metallic sheen. They were brittle and easy to break with a hammer into smaller pieces. Some of these fragments were ground in an agate mortar with a pestle for powder X-ray diffraction measurements, while others were mounted in epoxy and polished to yield flat surfaces for wavelength dispersive X-ray spectroscopy (WDS) measurements. In addition, prismatic crystals with smooth surfaces were identified in the products for examination with single crystal X-ray diffraction.

The powder X-ray diffraction pattern collected for our initial sample is shown in Figure 2a, where a complicated series of relatively broad peaks can be discerned from the background. Most of the peaks can be assigned to the hexagonal structure of the new compound Y_13_Ag_42.7_Zn_29.7_ described below_._ Additional peaks can be attributed to the binary YAg_2_ phase, as well as YAgSi (perhaps reflecting a reaction with the fused silica tube used as a container). There are a few minor peaks which could not be assigned to the elemental starting materials or known binary Y–Ag, Y–Zn, or Ag–Zn phases, suggesting that other new phases may be present as side products.

**Figure 2:**
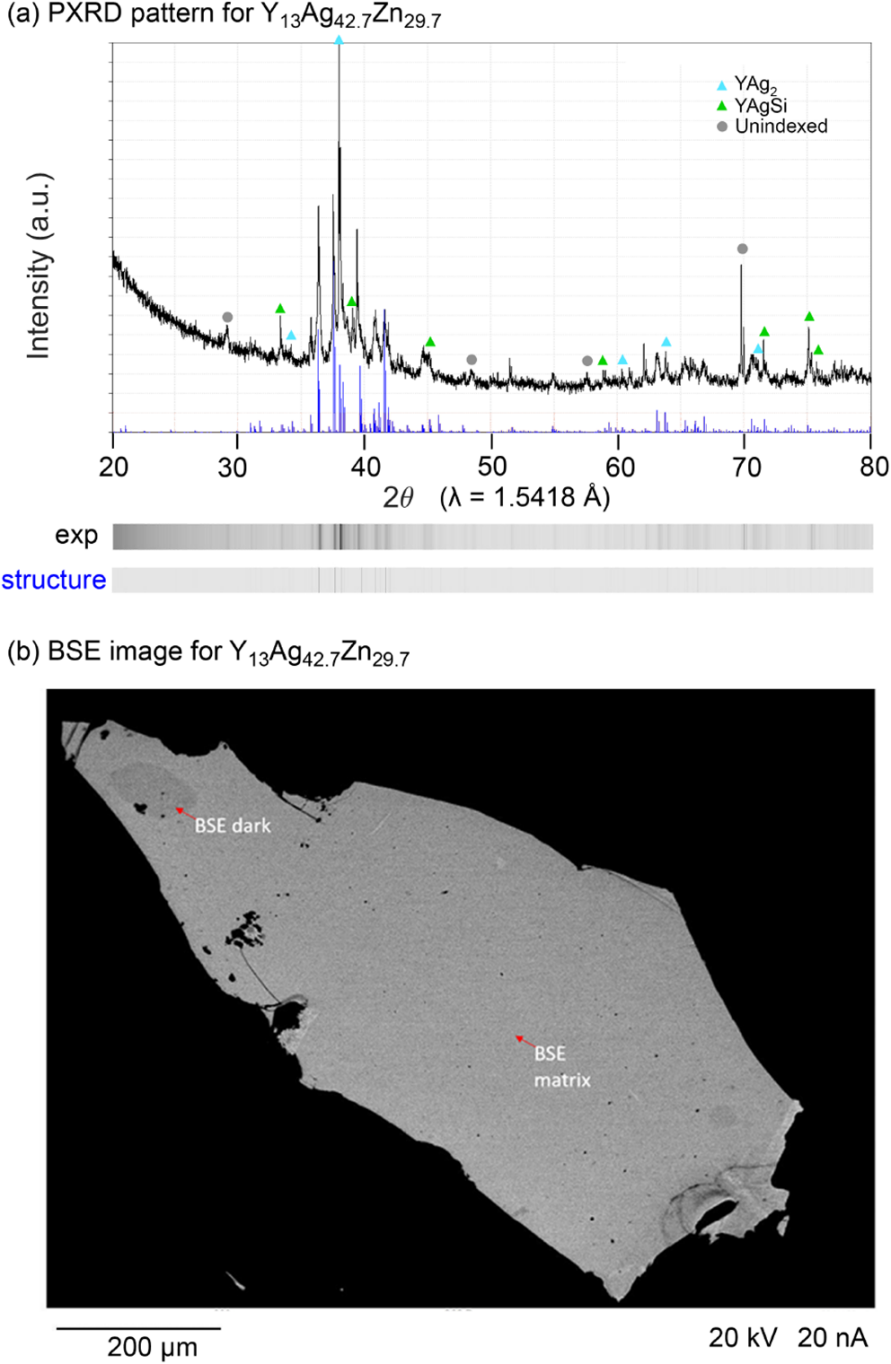
Phase analysis for a sample containing Y_13_Ag_42.7_Zn_29.7_. (a) Power X-ray diffraction pattern (black) with the calculated pattern (blue) from the refined structure. At the bottom, film strip representations are given of the experimental pattern as well as the simulated pattern for Y_13_Ag_42.7_Zn_29.7_. Peaks attributed to the impurities YAg_2_ and YAgSi, as well as those that remained unindexed, are marked. (b) SEM-BSE image of a polished fragment of the same sample, showing the different phases in the gray scale contrast. The matrix phase is assigned to Y_13_Ag_42.7_Zn_29.7_ based on WDS measurements, while the dark phase is likely a variant with a slightly lower Ag/Zn ratio.

Following our identification of Y_13_Ag_42.7_Zn_29.7_ through single crystal analysis (see below), we also attempted to produce a phase-pure sample through a stoichiometric synthesis. When the same temperature profile was used, however, only the unreacted elements were obtained. The product was then resealed in an evacuated fused silica tube and subjected to a more intense heat treatment: 800 °C for 24 h to initiate the reaction, and then 1000 °C for 168 h. Finally, the sample was cooled to ambient temperature at a rate of 50 °C/hour. The final product was comparable in its appearance and brittleness to that of our initial synthesis. From its powder X-ray diffraction pattern, Y_13_Ag_42.7_Zn_29.7_ again appears to be a prominent phase, though peaks assignable to YAgSi, and Y_2_O_3_ were also noted, along with three peaks that are unindexed. The latter two side products may indicate enhanced reaction with the fused silica tube (SiO_2_) at the higher temperatures used here.

A phase analysis with scanning electron microscopy-back scattered electron (SEM-BSE) coupled with WDS measurements was carried out on the sample corresponding to the powder pattern in Figure 2a. The SEM-BSE images reveal two different phases, labeled matrix and dark, with the former being the majority component (Figure 2b), and as well as a eutectic decomposition product (shown in the Supporting Information) that perhaps gives rise to the unindexed peaks in the powder pattern. The WDS composition for the matrix phase is Y_15.1(1)_Ag_50.1(3)_Zn_34.8(2)_ (40 measurement points). When normalized to 13 Y atoms formula unit, the resulting formula of Y_13_Ag_43.1(6)_Zn_30.0(3)_ matches the Y_13_Ag_42.7_Zn_29.7_ composition refined from the single crystal X-ray diffraction data to within 3 times the standard deviation.

The WDS composition for the minority dark phase is Y_15.7(2)_Ag_48.0(2)_Zn_36.3(2)_ (40 measurement points), with slightly higher Zn and lower Ag content than the title compound. This phase may represent a structural variant. The sharp boundary between the phases suggests that the target phase is saturated with respect to Zn content. WDS measurements were also taken on the eutectic decomposition 2-phase regions. However, the standard deviation was too high to give conclusive compositions.

### 3.2 Structure Solution and Refinements

The diffraction pattern of a single crystal picked from the Y–Ag–Zn product is indexable with a hexagonal unit cell with *a* = 19.760(7) Å and *c* = 9.079(3) Å, with a Laue symmetry and lack of systematic absences suggesting the space group, *P*6/*mmm* (Table 1). From these observations, the Ta_13_Co_40_Si_31_ type emerges as a potential candidate for the structure.^19–23^ The structure solution was performed with the charge-flipping algorithm, which confirmed the space group assignment. The refinement process quickly converged on a structural framework similar to that of the Ta_13_Co_40_Si_31_ type.

However, while other compounds in this type have been noted to exhibit some localized disorder, in this structure positional disorder spreads throughout the *z* = 0 layers (Figure 3), a feature that was evident even in the original electron density map generated by the charge-flipping algorithm. In addition, channels along **c** occur within the structure in which partial occupancies appear to correlate with elongated density features in the neighboring positions (Figure 4).

**Figure 3:**
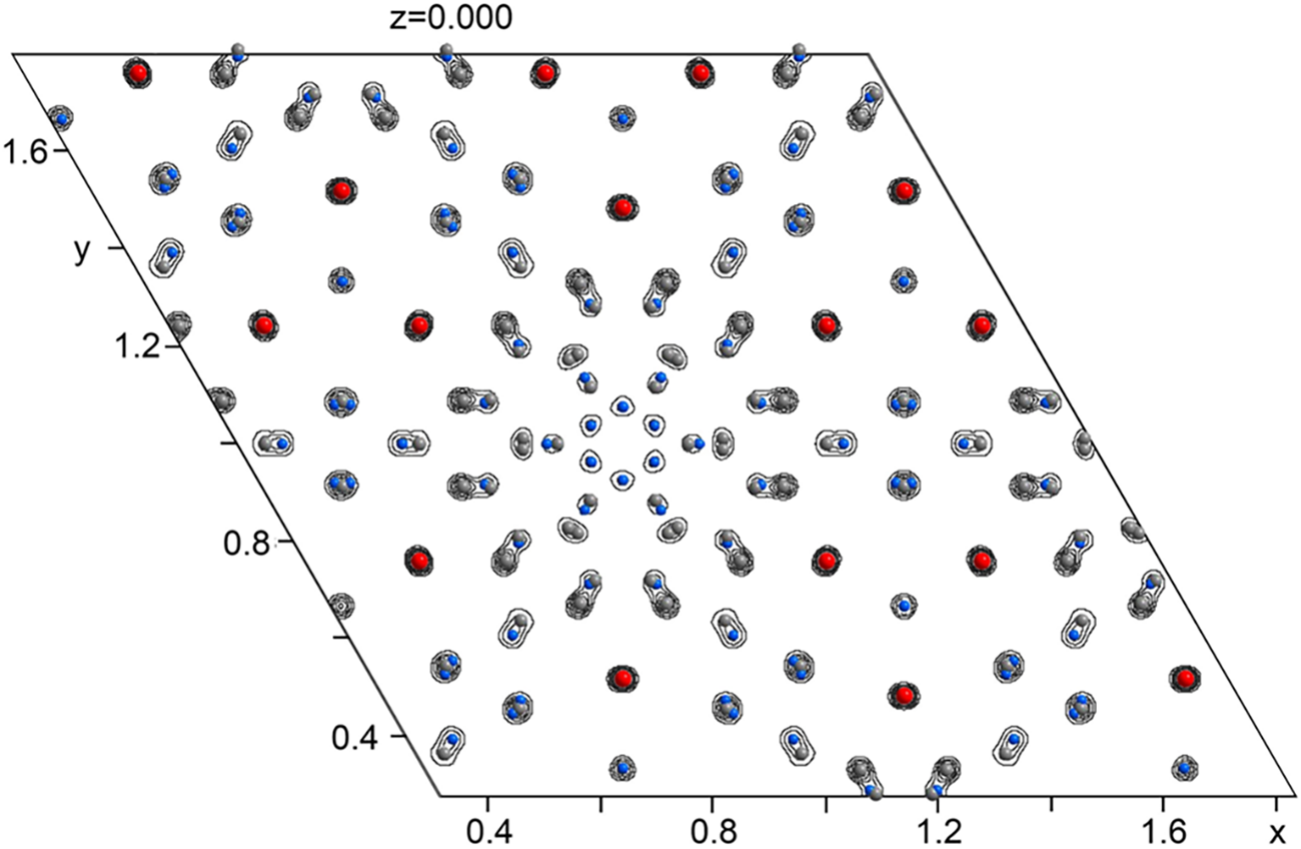
Fourier electron density contour for the disordered, *z* = 0.0 layer. The density contours are overlaid with the refined atomic positions. Y: red. Ag: gray. Zn: blue.

**Figure 4:**
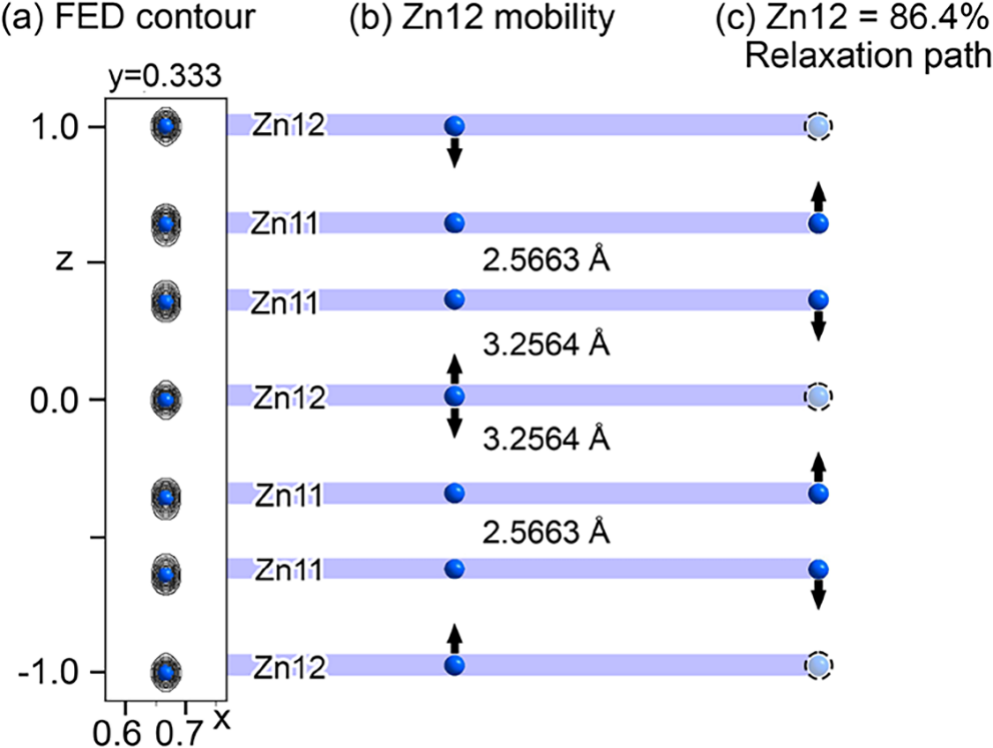
Disorder within Y_13_Ag_42.7_Zn_29.7_’s EuMg_5_-type channels. (a) Fourier electron density (FED) contour for the Zn sites (Zn11 and Zn12) in the channel, with elongated shapes. (b) The long Zn11—Zn12 interatomic distances create a large space for the Zn12 site to exhibit positional flexibility. (c) Elongation of the Zn11 features can be attributed to relaxations in response to vacancies on the Zn12 site (site occupancy = 86.1%).

After we noted the absence of satellite reflections in the diffraction patterns and attempts at refinement in lower space group symmetries did not lead to a reasonable model with greater order, we concluded that this disorder is an integral part of the crystal structure. To model it, we employed multiple split sites, mixed Ag/Zn sites, and a partially occupied Zn site (in the channel). With this approach, a converged refinement with reasonable R-factors and a shallow Fourier difference map was obtained with a composition that matched closely that from the WDS measurements.

One may wonder the degree to which disorder in the Y_13_Ag_42.7_Zn_29.7_ arises from static displacements of the atoms from average positions, as compared to vibrational effects. To investigate this question, we collected a single crystal X-ray data set at *T* = 100 K for comparison with the room temperature structure. The corresponding Fourier density map for the *z* = 0 layer is shown in the Supporting Information. It exhibits many of the same features as seen in Figure 3, but two sites show less radial smearing of the density toward and away from the closest hexagonal axis. The vibrations of these sites along these directions thus appear to be particularly enhanced by thermal energy.

### 3.3 Structure Description

As in several other complex intermetallic phases,^44–47^ the crystal structure of Y_13_Ag_42.7_Zn_29.7_ can be divided into a positionally ordered framework and disordered regions. Let’s start with the former as it provides the context in which the disorder arises. The Y_13_Ag_42.7_Zn_29.7_’s ordered framework consists mostly of three atomic layers at *z* = 0.25, 0.5, and 0.75 (Figure 5). The majority of these atoms trace out discs of Y-filled double hexagonal prisms, representing truncated sheets of the CaCu_5_ type. These blocks, in turn, stack along the *c*-axis separated from each other by a disordered layer of atoms, reminiscent of the division of the CaCu_5_ type into slabs separated by misfit layers of atoms in the CaPd_5+*x*_ structure. We will thus refer to the resulting columns as based on the CaPd_5+*x*_ type, though strictly speaking the CaCu_5_-type slabs in the latter are offset from each other rather than stacked in an eclipsed way.

**Figure 5:**
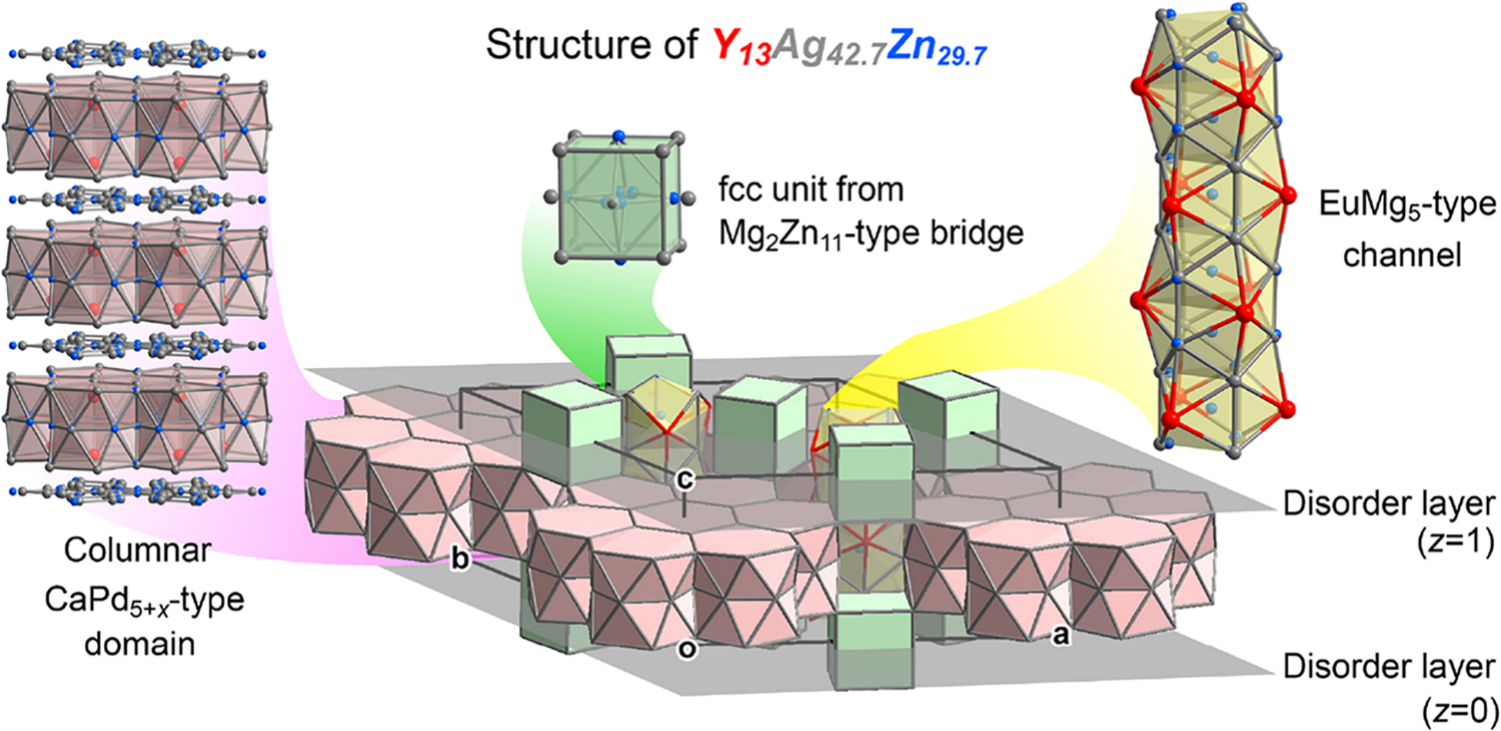
Overview of the structure of Y_13_Ag_42.7_Zn_29.7_. Separated by layers of disordered atoms at *z* = 0,1, discs derived from the CaPd_5+*x*_ type are joined into a hexagonal network through bridges of the Mg_2_Zn_11_ type (visible here just by fcc units derived from that parent structure). Though triangular gaps in this arrangement run channels derived from the EuMg_5_ type. The CaPd_5+*x*_-based discs stack along **c** to form columnar domains resembling this parent structure.

Within the *ab*-plane, the neighboring CaCu_5_-type hexagonal discs are bridged by unit-cell-sized cubes of the fcc structure (subject to some disorder where they cross the *z* = 0,1 layers), which share opposite edges of their square faces with the two discs. The resulting configuration of pairs of hexagonal antiprisms alternating with intermediary fcc units is, in fact, a feature of a second intermetallic structure type, the Mg_2_Zn_11_ type. Each disc is thus linked to six neighbors within the *ab*-plane by linear connecting regions of the Mg_2_Zn_11_ type. A similar combination of the CaPd_5+*x*_ and Mg_2_Zn_11_ type underlies the structures of YAg_2.79_Zn_2.80_, YAg_2.44_Zn_3.17_, and YAg_2.71_Zn_2.71_,^18^ though in those cases a lamellar morphology is observed.

This difference from these earlier structures can be connected to the presence of a third geometrical element: a tube of atoms that passes through the triangular gaps between the CaPd_5+*x*_-based columns. They consist of a stack of tricapped trigonal prisms alternating with flattened octahedra, with the interior space exhibiting a disordered occupation pattern. These channels are derived from the EuMg_5_-type framework,^30^ as adopted by the compound YZn_5+*x*_ in the Y–Ag–Zn system,^48,49^ and are notable for the tendency of their occupants to form disordered or modulated patterns.^50–53^ As we will see below, these channels in fact form the cores of larger domains of the EuMg_5_ type that play a key role in shaping the structure.

### 3.4 The Disordered *z* = 0 Layer

As we illustrated in Figure 3, the *z* = 0 layer separating the CaCu_5_-type hexagonal discs is plagued with disorder, including split sites and other partially occupied positions that are too close to each other to be simultaneously filled. Within this seeming chaos, however, potential patterns of local order can be discerned. We begin at the high symmetry point (0,0,0) resting just above and below the centers of CaCu_5_-type discs (Figure 6a). Surrounding this point is a hexagon of atomic positions representing the orbit of the Zn15 site, with the distances between neighboring Zn15 positions being too short to be occupied at the same time.

**Figure 6:**
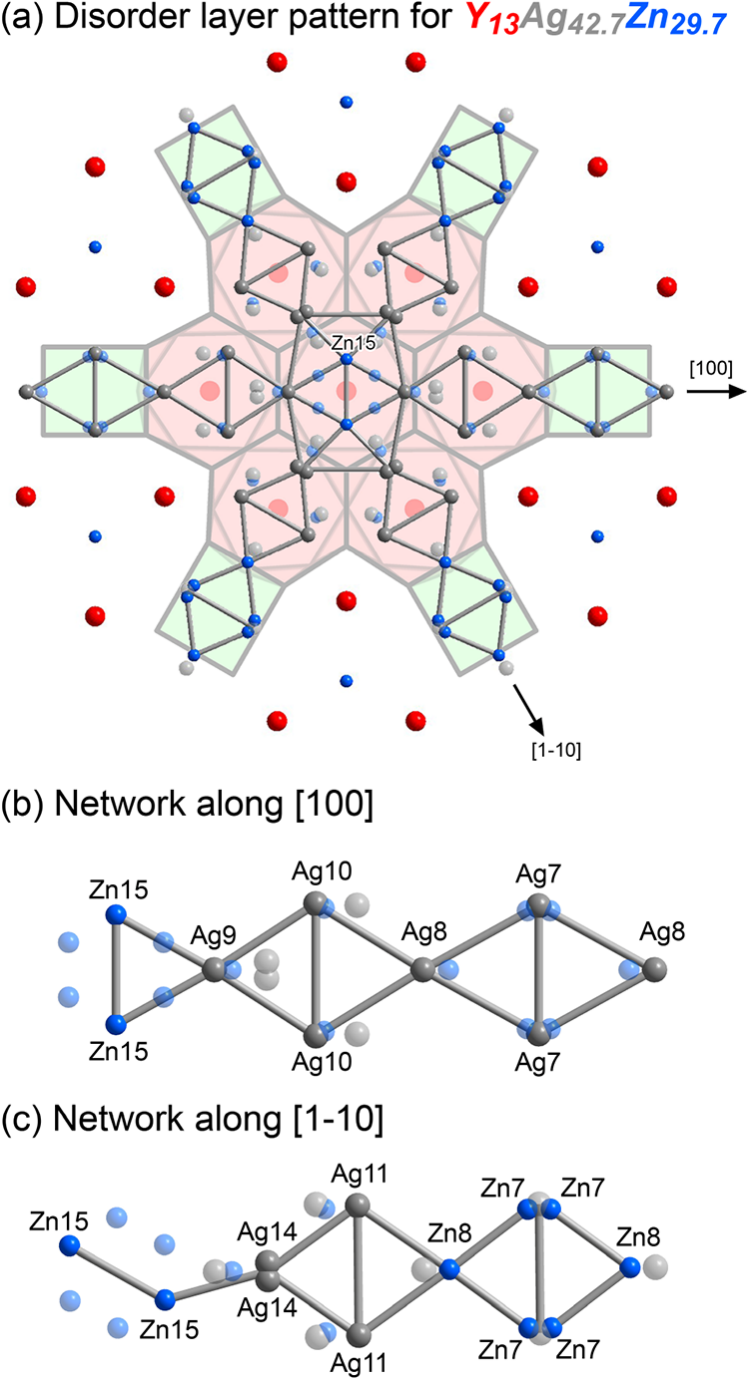
Potential local arrangements in the disordered *z* = 0 layer. (a) Snowflake-like configuration seeded by one of three possible Zn15 dumbbell orientations. Unused atomic positions are shown with partial transparency. (b) Close-up view with site labels for one branch of the pattern extended along the [100] direction. (c) Analogous view for a branch propagating along the [1–10] direction.

Similar disordered hexagons are observed in the Gd_14_Ag_51_ type^54–58^ (as adopted by Y_14_Ag_39.3_Zn_12.1_ in this system),^59^ where the situation can be solved by resolving the hexagon into two partially occupied triangles. In this case, though, the only physically reasonable interatomic distances are between atoms on opposite sides of the hexagon, with this Zn15–Zn15 distance being 2.56 Å. As such, the Zn15 position can be interpreted as representing a Zn dumbbell in three possible orientations. This scenario is illustrated in Figure 6a with two of the 6 positions being plotted with solid spheres, while the remaining ones are shown with partial transparency.

Once one of these dumbbells is chosen, we can work outward to find neighbors at allowable distances. Selecting the vertically oriented dumbbell, we find that it can be bridged on opposite sides by atoms at the Ag9 site (or its Zn counterpart Zn9), creating a rhombus. Each of the rhombus’ edges are bridged by the split Ag14 position, completing a distorted hexagon around the dumbbell.

From each corner, a string of two vertex-sharing rhombi extends outward to sample different selections of the remaining sites, depending on whether the initial corner is closer (Figure 6b) or further (Figure 6c) from the origin. The overall result is a snowflake-like pattern of atoms with orthorhombic local symmetry, stemming from the central dumbbell’s breaking of the crystallographic 6-fold axis. Disorder arises from the three possible orientations of this pattern, as well as variations in the Zn/Ag occupation of some of the sites, as in Zn7 vs Ag7.

These patterns influence the coordination environments of the Y atoms resting near the surfaces of the double hexagonal antiprisms above and below. The Y atoms become capped with either a (Ag/Zn) dumbbell or triangle depending on how the rhombi align with their positions in the *ab*-plane. The range of cation coordination environments mirrors those of the Ca atoms in CaPd_5+*x*_,^24^ albeit without the long-range order created by an incommensurate modulation.

### 3.5 EuMg_5+*x*_ Type Channel

A second region of disorder in the structure of Y_13_Ag_42.7_Zn_29.7_ is its EuMg_5_-type channels, whose occupants are modeled with the Zn11 and Zn12 positions (Figure 4). The Zn12 position is relatively isolated, being in the center of a tricapped trigonal prism, while the Zn11 atoms occupy the top and bottom triangular faces of the neighboring tricapped trigonal prisms to form dimers. The resulting interatomic distances between the occupants along the channel are rather uneven, with Zn11–Zn11 distances of 2.57 Å being within the normal range, but the Zn11–Zn12 distances being much longer at 3.26 Å. The Fourier electron density features for these sites are elongated along *z*, suggestive of variable positions within the channel.

The variability of the *z* position for the Zn11 sites can be at least partly understood by partial occupancy for Zn12 site (occ = 86.1%, not surprising considering its placement within the *z* = 0 layer). When no atom is present at the Zn12 site, the neighboring Zn11 atoms would be expected to move somewhat toward the vacancy to better equilibrize the local packing density. Elongation of the Zn12 Fourier density features, meanwhile, may reflect the long distances to the surrounding atoms along the channel.

### 3.6 Domain Interpenetration Analyzed with the *GrowDomain* Program

In our structural description above, we have seen how the crystal structure of Y_13_Ag_42.7_Zn_29.7_ contains fragments of several parent structures, including the CaPd_5+*x*_, Mg_2_Zn_11_, and EuMg_5_ types, merged into a complex arrangement. Let’s now consider whether this arrangement can be understood using the Interface Nucleus Approach. In this model, interfaces between parent structure domains are hypothesized to be stabilized by CP relief within regions of atoms shared by the domains, i.e., can be assigned to either domain. A key step in testing this idea is to explore the extent to which the different parent structure domains interpenetrate each other to create such intermediate regions. In practice this process can be challenging to carry out by structural inspection, as it entails going through a complex structure atom-by-atom and correlating each position with those expected for the continuation of the various domains present. In this section, we will illustrate how this process can be carried out in a computer-aided fashion using the program *GrowDomain*.

*GrowDomain* maps parallels between two crystal structures, such that atoms of a complex structure can be assigned to domains of a reference structure. It begins with atomic positions and connectivity graphs, generated with the *ToposPro* program,^42^ for a shared motif that is extracted from the two structures to act as a domain seed. The program then determines the isomorphism between the graphs and refines a transformation matrix to maximally superimpose the corresponding atomic positions onto each other. Once the structures are aligned and scaled accordingly, the program searches the surrounding of the domain seeds to find additional atoms that are nearly coincident between the complex and reference structures. Any atoms thus identified are added to the table of mapped atoms, and the process returns to the refinement of the transformation matrix. This cycle is repeated until no new corresponding atoms can be identified.

In Figure 7, we illustrate the results of this process, beginning with the most obvious structural motifs in Y_13_Ag_42.7_Zn_29.7_, the disc-shaped units derived from the CaPd_5+*x*_ structure. We extract the central double hexagonal antiprism from this structure and a counterpart from a simplified version of CaPd_5+*x*_ as a seed for the matching and apply *GrowDomain* to assign additional atoms to the domain. The process converges on the fifth search cycle, leading to the positions shown in yellow in Figure 7a. These points include all of the atoms of the CaPd_5+*x*_-type hexagonal disc that we presented in our structural description, but also capture additional atoms, which appear as a concentric ring around the disc when looking down the *c*-axis.

**Figure 7:**
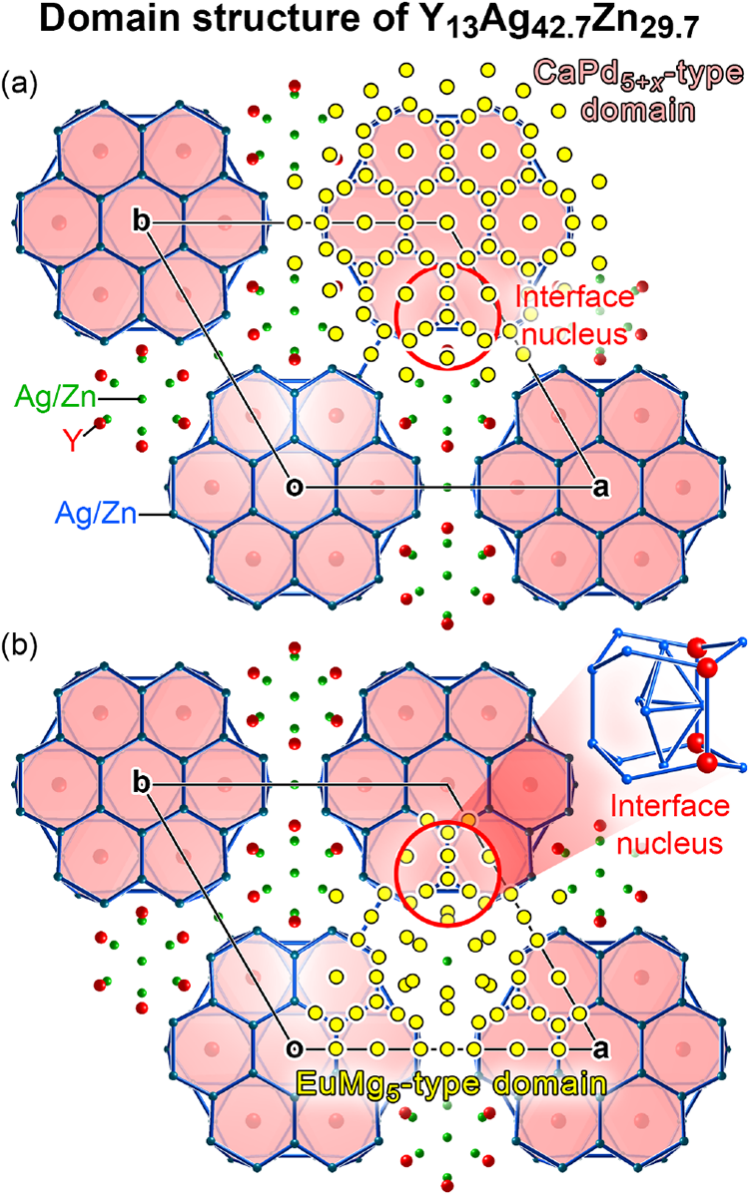
Parent structure domains within the framework of positionally ordered atoms in Y_13_Ag_42.7_Zn_29.7_, as obtained from *GrowDomain*. (a) The ordered framework with the atomic positions for a domain of the CaPd_5+x_ type indicated in yellow. (b) The corresponding image for a EuMg_5_-type domain. In both images, a red circle highlights a region of atoms that is shared by the two domains, representing a possible interface nucleus.

The expanded domain extends from the cell corner to halfway along **a**, **b**, and **a**+**b**, such that neighboring domains within the *ab*-plane share atoms at their edges. Now almost all of the atoms of ordered framework can be assigned to one (or more) of these regions. In this way, much of the structure can be derived by beginning with CaPd_5+*x*_-type sheets (stacked in an eclipsed fashion), cutting it into columns and rejoining the columns to make a hexagonal framework with shared atoms at their edges.

The exceptions are localized around specific points: the EuMg_5_-type channels we identified as running along **c** and passing between the CaPd_5+*x*_-type regions. Do these units of the EuMg_5_-type form the cores of larger domains of this type in Y_13_Ag_42.7_Zn_29.7_? This possibility can be explored by entering the ordered framework of Y_13_Ag_42.7_Zn_29.7_ and the EuMg_5_ structure type into *GrowDomain*, and using a basic element of the EuMg_5_-type channel, a tricapped trigonal prism (minus the central atom, which is subject to disorder in both structures), as a domain seed. The results are overlaid on the ordered framework of Y_13_Ag_42.7_Zn_29.7_ in Figure 7b: a triangular region that penetrates deeply into regions claimed by neighboring CaPd_5+*x*_-based domains, nearly to their centers at the unit cell corners.

The substantial overlap between the CaPd_5+*x*_-based and EuMg_5_-type domains is in line with the Interface Nucleus Approach to modular intermetallic compounds. Notably, among the atoms in the shared regions is a full unit of the TCP unit characteristic of the EuMg_5_ type, a trigonal bipyramid enclosed by an iceane-type cage (Figure 8), which is derivable from the MgZn_2_ type. As a recurring motif across several structures, this unit is an excellent candidate for an interface nucleus, and we will explore the CP features it exhibits in the CaPd_5+*x*_- and EuMg_5_-type parent structures below.

**Figure 8:**
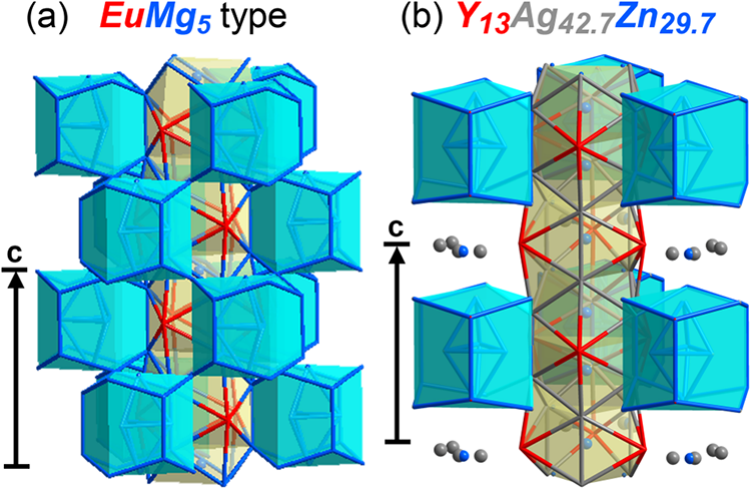
Comparison of the surroundings of the EuMg_5_-type channels in the (a) EuMg_5_ type itself and (b) Y_13_Ag_42.7_Zn_29.7_. In the original EuMg_5_ type, the channel is ensheathed by triangles of TCP units (turquoise) that stack along **c** with staggered configurations. In the Y_13_Ag_42.7_Zn_29.7_ structure, every other triangle of TCP units is missing, leading to gaps in the correspondence with the EuMg_5_ type. These gaps are centered on the *z* = 0,1 disordered layers.

A third parent structure that contributes to the ordered framework of Y_13_Ag_42.7_Zn_29.7_ is the Mg_2_Zn_11_ type. As shown in the Supporting Information, a *GrowDomain* analysis reveals a domain of this structure that encompasses a significant fraction of the cell volume. As we have discussed interfaces between such CaPd_5+*x*_ and Mg_2_Zn_11_ types previously,^18^ we will focus here on the CaPd_5+*x*_ and EuMg_5+*x*_ domains, which together form a fairly complete description of the ordered framework.

### 3.7 CP Relief at the Domain Boundaries

In the process of mapping the atomic coordinates of Y_13_Ag_42.7_Zn_29.7_ to its parent structures, *GrowDomain* generated transformation matrices that allow us to superimpose the domains in the complex structures with the corresponding sections of the parent structures. This capability opens the opportunity to test in detail the hypothesis that CP issues in the parent structures drive their intergrowth. This picture predicts that the atoms at the domain edges should be displaced in ways that relieve the CP issues in the original parent structures.

In Figure 9a, we illustrate this approach with a CaPd_5+*x*_-based domain in Y_13_Ag_42.7_Zn_29.7_. We show the atomic positions from the simplified CaPd_5+*x*_-type parent structure that have counterparts in a domain of Y_13_Ag_42.7_Zn_29.7_. For those atoms that have a relatively tight fit to positions in the complex structure (mismatch below 0.46 Å, a value that distinguishes the fidelities to the ideal structure of the atoms at the interior of the domain from those near the exteriors), we display their CP features, calculated for a YAg_5_ model of the parent structure, using the standard conventions: each atom is surrounded by a radial surface with the distance of a point on the surface to the atomic center being proportional to the magnitude of the sum of the pressure contributions experienced by the atom along that direction. The sign of the pressure is given by the color of the surface: black for negative pressure (overly sparse packing), white for positive pressure (overly dense packing). In this case, tensions we have noted before for this structure^24^ are evident, with positive CPs between the Ag atoms, with the lobes in kagome nets being particularly dominant, holding open the coordination environments of the cations with negative CPs.

**Figure 9:**
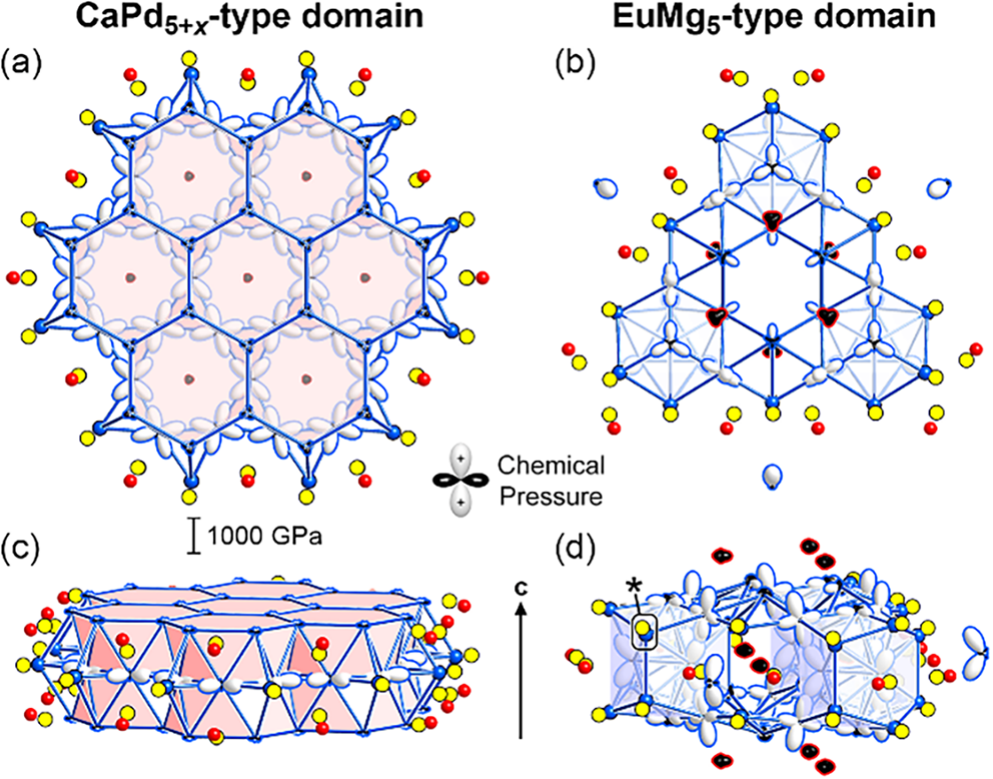
Correlation of atomic displacements at domain edges in Y_13_Ag_42.7_Zn_29.7_ with the CP schemes of the parent structures. (a) Top-down view of a CaCu_5_-type disc in a CaPd_5+x_-based domain. CP features calculated for a hypothetical YAg_5_ parent are shown overlaid on positions of a simplified version of the CaPd_5+*x*_ structure where the mismatches to the transformed positions of the Y_13_Ag_42.7_Zn_29.7_ are less than 0.46 Å. For positions with greater mismatch, the parent structure positions are shown with blue (Pd-type positions) or red (Ca-type positions) spheres while their counterparts from Y_13_Ag_42.7_Zn_29.7_ are plotted for comparison in yellow. (b) Analogous plot for the EuMg_5_-type domain with the CP lobes being shown for sites with mismatches less than 0.53 Å. (c)–(d) Side views of the configurations in (a) and (b), respectively. An asterisk in (d) marks a site whose displacement in Y_13_Ag_42.7_Zn_29.7_ relative to the expected position is not accounted for by the CP scheme of the EuMg_5_ type. The mismatch cutoffs of 0.46 and 0.53 Å for the CaPd_5+x_-based and EuMg_5_-type domains are chosen to draw a distinction between their interior and exterior regions. The CP scalebar below (a) relates the sizes of the lobes in all for panels to the CP magnitudes.

For the remaining atoms, we overlay them instead with the position of the corresponding atom in the complex structure using yellow spheres. These more displaced atoms are restricted to the outer regions of the domain and their positions in the complex structure are responsive to the packing issues in the parent. The Y atoms at the boundary are drawn inward within the *ab*-plane toward the domain to achieve closer contacts with atoms projecting negative CP features toward them. Conversely, the Ag atoms appear to be driven outward in response to positive CP lobes directed toward them.

Analogous effects are evident in the corresponding analysis of the EuMg_5+*x*_ domains (Figure 9b). The displacements of the atoms in Y_13_Ag_42.7_Zn_29.7_ relative to idealized positions in the EuMg_5_ type are concentrated on the exterior of the region. In most cases, each displaced atom moves away from positive CP lobes pointing at them from the interior or toward negative CP features. The exceptions are the atoms at the 3-fold connected vertices of the iceane cages on the outskirts of the domains (most visible in Figure 9d). These move outward despite the absence of any positive pressure pushing them in that direction; instead, small negative CPs appear to be pulling them inward. In fact, these sites switch from Ag in the EuMg_5_-type YAg_5_ parent structure to Y in Y_13_Ag_42.7_Zn_29.7_. The large size of the Y atoms is consistent with this outward shift and negative pressures directed toward the site from the interior of the domain. Altogether, atomic displacements from the idealized parent structure suggest that the Y_13_Ag_42.7_Zn_29.7_ structure has taken advantage of the domain boundary as an opportunity for the release of CP.

### 3.8 CP Complementarity at Interface Nuclei

In the last section, we saw how the edges of both the CaPd_5+*x*_-based and EuMg_5_-type domains exhibit atomic displacements in accordance with preferences of the atomic packing tensions of the parent structures. That the domain interfaces can simultaneously accommodate the preferred relaxations of both parent structures is suggestive of complementarity between their CP features. The potential for such compatibility can be investigated more directly using the *CP*_interface_ function, which captures how the CPs are directed toward a chosen surface (qualitatively representing a CP flux), such as a planar interface.^40^

In this case, we use as surfaces the convex hulls of the iceane-based TCP units in the two parent structures, calculate the *CP*_interface_ functions for each surface, and compare the *CP*_interface_ function values at corresponding points (Figure 10). The function for the simplified CaPd_5+*x*_ type has a simple form, with pairs of positive pressure spots appearing at intervals of 120° along the middle of the barrel-like surface. These features reflect positive Ag–Ag CPs within the kagome nets at this height in the structure. The remainder of the surface is essentially neutral.

**Figure 10:**
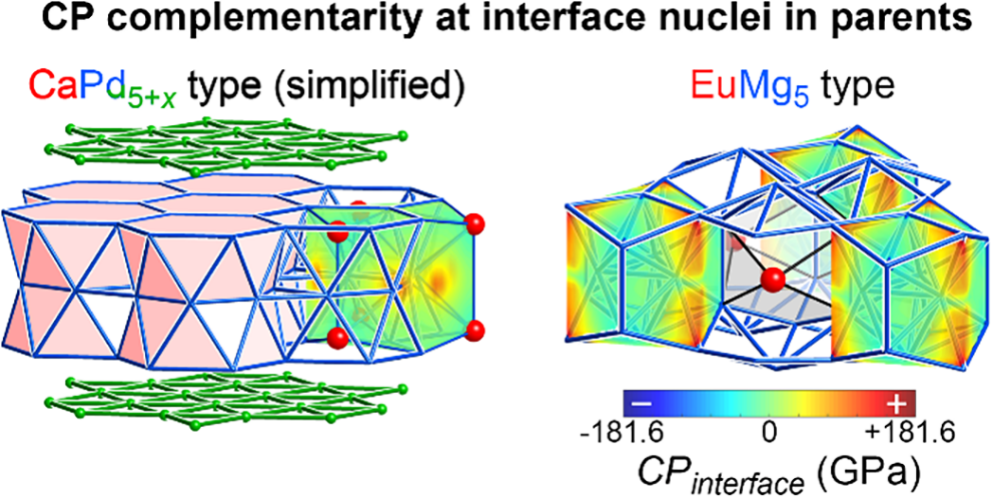
*CP*_*interface*_ function plots, mapping the packing tensions between an interface nucleus and its surroundings, for the icecane-cage-bounded TCP units in hypothetical YAg_5_ compounds adopting a simplified version of the CaPd_5+*x*_ type and the EuMg_5_ type.

The *CP*_interface_ function for the interface nucleus in the EuMg_5_ type displays a similar contrast between points of intense positive CPs and a shallower background. This time, though, the intense features are placed above and below the midline, rather than right along it. If we imagine the formation of an interface between the two structure types as creating an intermediate environment for the interface nucleus that tends to average their features, the staggering of their strong CPs sets up a favorable situation. The positive CPs that would reinforce each other in the propagation of the parent structures alone now interdigitate, reducing the buildup of positive CP within the structure. An analogous scenario is encountered in the intergrowth of the Cr_3_Si- and Al_3_Zr_4_-types to form the σ-phase structure.^40^

As we presented previously for the lamellar intergrowths in the Y–Ag–Zn, similar CP complementarity arises at the interface nuclei for the CaPd_5+*x*_ *-* and Mg_2_Zn_17_-type parent structures.^58^ In this manner, the 3-fold intergrowth of the CaPd_5+*x*_, Mg_2_Zn_17_, and EuMg_5_ types has opportunities for CP relief at two of the three potential interface types. In the Supporting Information, we also consider the remaining combination: the boundary between the Mg_2_Zn_17_- and EuMg_5_-type regions. A shared motif can indeed be identified, consisting of a single distorted hexagonal antiprism. The *CP*_*interface*_ functions for this unit in the two parent structures, however, show very similar features, and only minor CP values compared to those in Figure 10. Little potential for CP relief (or need for it) would be present this interface, suggesting that it arises from geometrical necessity rather than as a stabilizing feature.

### 3.9 Templated Framework in the Y_13_Ag_42.7_Zn_29.7_ Structure

The ordered framework of Y_13_Ag_42.7_Zn_29.7_ continues a recurring theme in modular intermetallic structures: the templating of domain morphology by the distribution of interface nuclei in the parent structures. Within the simplified CaPd_5+*x*_ structure type, the TCP interface nuclei are distributed in a hexagonal fashion along CaCu_5_-type sheets. The CaPd_5+x_-based discs in Y_13_Ag_42.7_Zn_29.7_ reflect this distribution, with each disc being decorated by a ring of six interface nuclei poised to merge with a compatible structure (Figure 11). Within the EuMg_5_ type, meanwhile, these units surround the EuMg_5_-type channels in a 3-fold, trigonal planar fashion at any given height along **c**.

**Figure 11:**
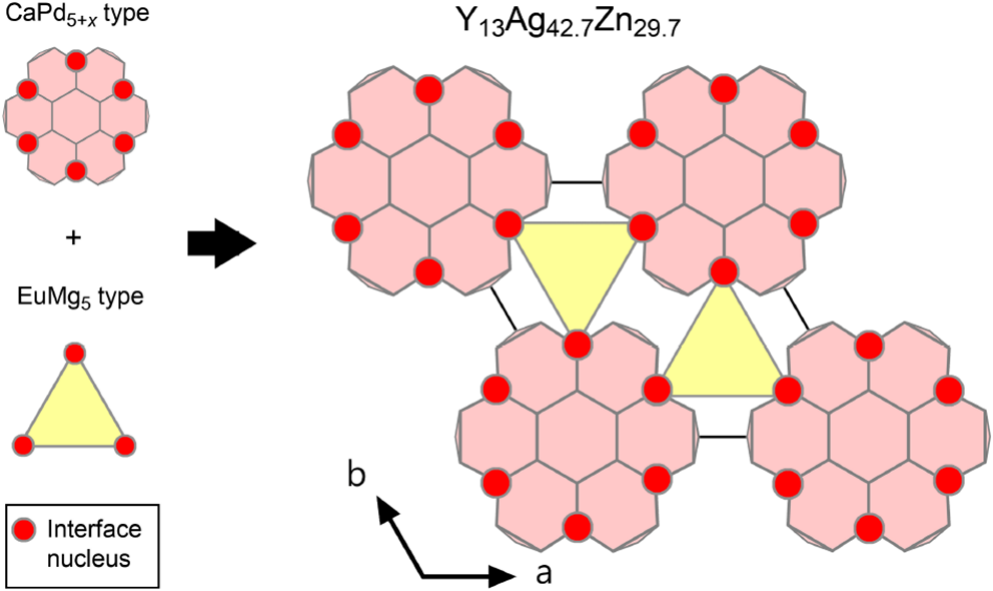
Schematic view of the templating of the Y_13_Ag_42.7_Zn_29.7_ structure’s ordered framework by the interface nucleus distributions in the CaPd_5+*x*_ and EuMg_5_ types.

If we consider the CaPd_5+*x*_-based discs as planar units with six open docking sites, and the EuMg_5_-type blocks as triangular units with three open docking sites, the observed domain morphology of Y_13_Ag_42.7_Zn_29.7_ allows for the two parent structures to meet at every docking site. Furthermore, the preference for the domains of the two types to join in an edge-on fashion, rather than say growing a EuMg_5_-type domain above or below a CaPd_5+*x*_-based disc can be understood from the nature of the CP complementarity at the interface nuclei (Figure 10). The strongest CPs have large components directed along the *ab*-plane, making changes to the structural arrangements in this plane the most effective for addressing the packing issues.

This picture of a templated architecture also helps account for the emergence of disordered layers largely separating the structure into sheets. Moving one atomic layer upward or downward from docked interface nuclei in Y_13_Ag_42.7_Zn_29.7_, a mismatch between the parent structures is encountered. In the CaPd_5+*x*_ type, essentially close-packed layers of transition metal atoms occur at these heights (though with a periodicity incommensurate with the remainder of the structure). In the EuMg_5_ type, on the other hand, another trefoil of interface nuclei is present, rotated by 60° from the ones above and below (see Figure 8a). The loss of coherence between the two parents in this layer makes the presence of disorder here understandable. In fact, the positional disorder extending over the whole layer represents an (impressive) example of a growing correlation between domain mismatch in modular intermetallics and positional disordered or weak ordered regions.

## 4 Conclusions

While understanding and predicting the structural chemistry of intermetallics are multifaceted challenges, principles for their modular arrangements are increasingly coming into focus. Here, we have presented the synthesis, structure determination, and analysis of a new compound, Y_13_Ag_42.7_Zn_29.7_, that realizes and elaborates the Interface Nucleus Approach for these architectures, in which modular structures are assembled from units that dock at shared groups of atoms with complementary CP features. Its structure consists of columnar domains derived from the CaPd_5+*x*_ type which are joined into a rod-packing by interpenetrating regions of the EuMg_5_ type. This arrangement is facilitated by complementary CP features at interface nuclei derived from the iceane-like motifs of the MgZn_2_ type. Finally, layers of extensive disorder arise at heights along the structure’s hexagonal *c*-axis where the coherence between the parents is lost.

These results affirm several recent conclusions derived from the interface nucleus approach to modular structures.^10,11,18^ Clear complementarity in the CP schemes of the parent structures is evident at the shared motif. In addition, a strong correlation arises between the directions of the key CP features and the orientation of the domain interfaces. Also as in other modular structures, the domain morphology is largely determined by the distributions of these units within the parent structures. Furthermore, the emergence of disorder in regions of mismatch or gaps in the modular architecture is consistent with other interface-nucleus-based structures. An open question for future research is the degree to which this picture can be applied or generalized beyond such intermetallics to the broader diversity of intergrowth structures in inorganic materials.^60–65^

The analysis of Y_13_Ag_42.7_Zn_29.7_ has also supported new developments in the Interface Nucleus Approach. The deep interpenetration of the domains calls for a computer-aided process to trace the parallels with the parent structures; the *GrowDomain* program was introduced to fill this role, which significantly simplifies the determination of the interface nuclei linking the domains. Along these lines, the capability of overlaying the atomic positions of Y_13_Ag_42.7_Zn_29.7_ with their counterparts in the parent structure elucidates how relaxations at the domain interfaces match the dictates of the CP schemes of the parent structures.

Another intriguing feature of Y_13_Ag_42.7_Zn_29.7_ is the evolution in domain structure relative to its more Ag-poor congeners YAg_2.79_Zn_2.80_, YAg_2.44_Zn_3.17_, and YAg_2.71_Zn_2.71_. These previously determined structures have layered domains, whereas in Y_13_Ag_42.7_Zn_29.7_ they adopt a hexagonal arrangement of columns. Structurally, this change in morphology is traced to the growth of EuMg_5_-type domains with increased Ag content, with the trigonal distribution of interface nuclei prompting a hexagonal pattern. Indeed, the walls of the EuMg_5_-type channels are enriched in Ag content relative to the rest of the structure (despite the fact that structures based on a EuMg_5_-type framework exist in the Y–Zn binary system).^48,49^ We are looking forward to probing how such compositional perturbations and elemental site preferences influence the relative stabilities of competing parent structures and the favorabilities of their intergrowth into larger-scale assemblies.

## Supplementary Material



## Notes

Compositional map showing the placement of the title compound in the context of other phases in the Y–Ag–Zn system; additional crystallographic tables; powder diffraction pattern for the product of the attempted stoichiometric synthesis of the title compound; cross-section for the Fourier density for a data set collected at T = 100 K; WDS data points; GrowDomain results for the Mg_2_Zn_11_ domains in the title compound; *CP*_interface_ functions for an interface nucleus for the Mg_2_Zn_11_-type/EuMg_5_-type domain boundaries; optimized geometries and total energies of hypothetical YAg_5_ compounds adopting a simplified version of the CaPd_5+*x*_ type and the EuMg_5_ type (PDF)
