# Supplementary material for: Interface Nucleus Templating of Modular Intermetallic Morphologies: Chemical Pressure Complementarity, Columnar Domains, and Complex Disorder in Y13Ag42.7Zn29.7
Source: Inorg Chem. 2025 Oct 25;64(44):21959–70. doi: 10.1021/acs.inorgchem.5c03538 (PMC12606709; doi:10.1021/acs.inorgchem.5c03538)
Supplement: Supplementary file 1 [file ic5c03538_si_001.pdf]

SUPPORTING INFORMATION

for

Interface Nucleus Templating of Modular Intermetallic Morphologies: Chemical Pressure Complementarity, Columnar Domains, and Complex Disorder in $Y_{13}Ag_{42.7}Zn_{29.7}$

Rie T. Fredrickson* and Daniel C. Fredrickson*

Department of Chemistry, University of Wisconsin—Madison,
1101 University Avenue, Madison, Wisconsin 53706, United States

*Corresponding authors. E-mail: rie.fredrickson@wisc.edu, danny@chem.wisc.edu

S1. Context for $Y_{13}Ag_{42.7}Zn_{29.7}$ in the Y-Ag-Zn System

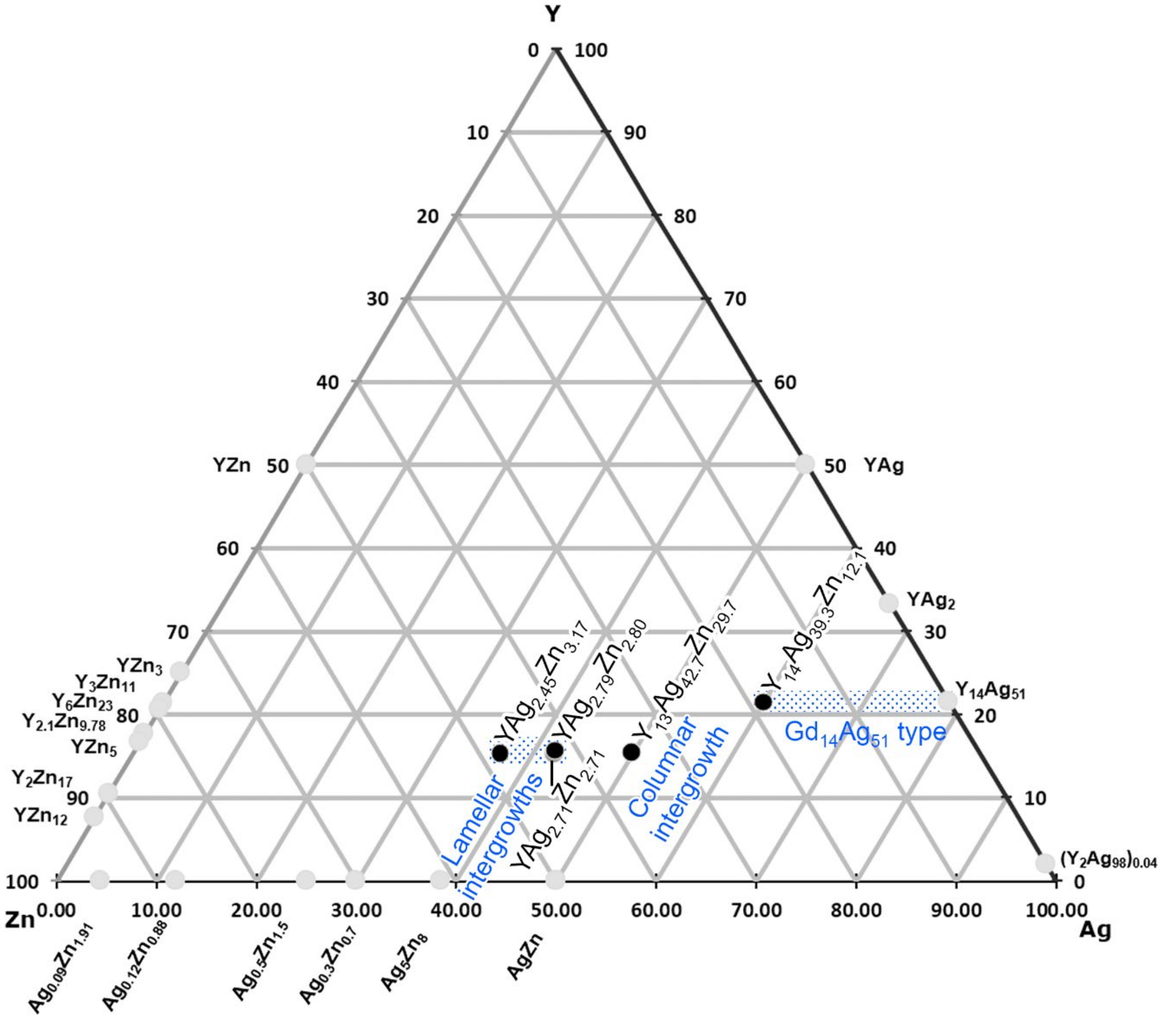

Figure S1. Schematic diagram of $Y_{13}Ag_{42.7}Zn_{29.7}$'s placement in the Y-Ag-Zn system relative to other ternary restructures previously investigated. Inferred compositional regions for $CaPd_{5+x}$-type/Mg_2Zn_{11}-type lamellar intergrowths and the $Gd_{14}Ag_{51}$ type are marked with blue stippling.

S2. Crystallographic Information Tables for $Y_{13}Ag_{42.7}Zn_{29.7}$

Tables are given describing the atomic coordinates, atomic displacement parameters, and selected refined atomic distances for the $Y_{13}Ag_{42.7}Zn_{29.7}$ structure.

Table S1. Refined atomic coordinates for the structure of $Y_{13}Ag_{42.7}Zn_{29.7}$.

Site	Element	Wyckoff position	*x*	*y*	*z*	U_{equiv}	Occupancy
Y1	Y	2*e*	0	0	0.30121(11)	0.0110(2)	1
Y2	Y	6*l*	0.758727(18)	0.51745(4)	0	0.0181(2)	1
Y3	Y	12*n*	0.27131(2)	0	0.29955(5)	0.01095(14)	1
Y4	Y	6*m*	0.577413(16)	0.422587(16)	1/2	0.01181(19)	1
Ag1	Ag	6*k*	0.58705(2)	0.58705(2)	1/2	0.01282(18)	0.921(7)
Zn1	Zn	6*k*	0.58705(2)	0.58705(2)	1/2	0.01282(18)	0.079(7)
Ag2	Ag	12*o*	0.821656(10)	0.64331(2)	0.24807(4)	0.01090(14)	0.665(5)
Zn2	Zn	12*o*	0.821656(10)	0.64331(2)	0.24807(4)	0.01090(14)	0.335(5)
Ag3	Ag	6*m*	0.866000(17)	0.73200(4)	1/2	0.0107(2)	0.217(7)
Zn3	Zn	6*m*	0.866000(17)	0.73200(4)	1/2	0.0107(2)	0.783(7)
Ag4	Ag	6*k*	0.86517(3)	0.86517(3)	1/2	0.0116(3)	0.113(7)
Zn4	Zn	6*k*	0.86517(3)	0.86517(3)	1/2	0.0116(3)	0.887(7)
Ag5	Ag	12*o*	0.74254(13)	0.4851(3)	0.3425(6)	0.0115(4)	0.788(5)
Zn5	Zn	12*o*	0.7452(8)	0.4903(16)	0.347(4)	0.0115(4)	0.211(5)
Ag6	Ag	12*o*	0.79128(9)	0.39564(4)	0.16811(14)	0.0154(3)	0.682(6)
Zn6	Zn	12*o*	0.7718(2)	0.38589(12)	0.1648(6)	0.0154(3)	0.318(6)
Ag7	Ag	6*l*	0.5436(3)	0.4564(3)	0	0.017(4)	0.416(8)
Zn7	Zn	12*p*	0.5264(8)	0.4455(15)	0	0.017(4)	0.292(4)
Ag8	Ag	6*j*	0.63729(14)	0.63729(14)	0	0.0232(6)	0.292(2)
Zn8	Zn	6*j*	0.60702(10)	0.60702(10)	0	0.0232(6)	0.649(4)
Ag9	Ag	6*j*	0.116(2)	0	0	0.016(4)	0.15(3)
Zn9	Zn	6*j*	0.134(3)	0	0	0.016(4)	0.12(4)
Ag10	Ag	12*p*	0.808(3)	0.718(4)	0	0.015(5)	0.103(13)
Zn10	Zn	12*p*	0.7956(8)	0.7115(14)	0	0.015(5)	0.346(13)
Ag11	Ag	12*p*	0.75897(9)	0.67080(11)	0	0.0192(5)	0.600(4)
Ag12	Ag	12*o*	0.909199(10)	0.81840(2)	0.23506(4)	0.01527(12)	1
Ag13	Ag	24*r*	0.652794(15)	0.551671(14)	0.22941(3)	0.01597(10)	1
Ag14	Ag	12*p*	0.8276(2)	0.8163(2)	0	0.0197(12)	0.220(3)
Zn11	Zn	4*h*	2/3	1/3	0.35867(15)	0.0308(3)	1
Zn12	Zn	2*c*	2/3	1/3	0	0.0226(5)	0.864(6)
Zn13	Zn	6*i*	1/2	1/2	0.26389(8)	0.0147(2)	1
Zn14	Zn	12*q*	0.72798(3)	0.59711(3)	1/2	0.00898(18)	1
Zn15	Zn	6*l*	0.07493(11)	0.03747(5)	0	0.0209(7)	0.408(3)

Table S2. Refined atomic displacement parameters for $Y_{13}Ag_{42.7}Zn_{29.7}$.

Site	U_{11}	U_{22}	U_{33}	U_{12}	U_{13}	U_{23}
Y1	0.0110(3)	0.0110(3)	0.0110(5)	0.00551(14)	0	0
Y2	0.0266(3)	0.0127(3)	0.0104(3)	0.00633(14)	0	0
Y3	0.01176(16)	0.00862(19)	0.0114(2)	0.00431(9)	-0.00008(13)	0
Y4	0.0108(2)	0.0108(2)	0.0136(3)	0.0052(2)	0	0
Ag1	0.0136(2)	0.0136(2)	0.0141(3)	0.00898(19)	0	0
Zn1	0.0136(2)	0.0136(2)	0.0141(3)	0.00898(19)	0	0
Ag2	0.01061(17)	0.0118(2)	0.0107(2)	0.00591(10)	-0.00021(6)	-0.00042(12)
Zn2	0.01061(17)	0.0118(2)	0.0107(2)	0.00591(10)	-0.00021(6)	-0.00042(12)
Ag3	0.0110(3)	0.0082(3)	0.0120(3)	0.00411(16)	0	0
Zn3	0.0110(3)	0.0082(3)	0.0120(3)	0.00411(16)	0	0
Ag4	0.0122(3)	0.0122(3)	0.0130(4)	0.0080(3)	0	0
Zn4	0.0122(3)	0.0122(3)	0.0130(4)	0.0080(3)	0	0
Ag5	0.0109(2)	0.0105(7)	0.0129(5)	0.0053(4)	-0.0007(2)	-0.0014(4)
Zn5	0.0109(2)	0.0105(7)	0.0129(5)	0.0053(4)	-0.0007(2)	-0.0014(4)
Ag6	0.0184(6)	0.0142(2)	0.0151(2)	0.0092(3)	-0.0015(4)	-0.0007(2)
Zn6	0.0184(6)	0.0142(2)	0.0151(2)	0.0092(3)	-0.0015(4)	-0.0007(2)
Ag7	0.015(5)	0.015(5)	0.0141(4)	0.002(5)	0	0
Zn7	0.015(5)	0.015(5)	0.0141(4)	0.002(5)	0	0
Zn8	0.0249(7)	0.0249(7)	0.0156(5)	0.0095(7)	0	0
Ag8	0.0249(7)	0.0249(7)	0.0156(5)	0.0095(7)	0	0
Ag9	0.015(8)	0.0160(13)	0.0187(13)	0.0080(6)	0	0
Zn9	0.015(8)	0.0160(13)	0.0187(13)	0.0080(6)	0	0
Ag10	0.015(7)	0.029(6)	0.0101(6)	0.018(7)	0	0
Zn10	0.015(7)	0.029(6)	0.0101(6)	0.018(7)	0	0
Ag11	0.0283(6)	0.0258(7)	0.0135(3)	0.0210(6)	0	0
Ag12	0.01420(13)	0.01612(17)	0.01613(18)	0.00806(9)	0.00165(6)	0.00331(12)
Ag13	0.01337(13)	0.01611(13)	0.01749(14)	0.00666(10)	-0.00190(9)	-0.00094(9)
Ag14	0.0218(14)	0.0211(18)	0.0169(6)	0.0113(14)	0	0
Zn11	0.0114(3)	0.0114(3)	0.0698(8)	0.00569(14)	0	0
Zn12	0.0122(6)	0.0122(6)	0.0434(11)	0.0061(3)	0	0
Zn13	0.0127(2)	0.0127(2)	0.0176(4)	0.0055(3)	0	0
Zn14	0.0084(2)	0.0098(2)	0.0093(2)	0.00507(18)	0	0
Zn15	0.0114(9)	0.0318(10)	0.0127(9)	0.0057(5)	0	0

Table S3. Selected interatomic distances for $Y_{13}Ag_{42.7}Zn_{29.7}$.

Site 1	Site 2	Distance (Å)	Multiplicity	Site 1	Site 2	Distance (Å)	Multiplicity
Y1	Y1	3.610(3)			Zn14	2.697(2)	
	Ag4/Zn4	3.218(3)	×2	Ag4/Zn4	Ag4/Zn4	2.664(2)	×2
	Ag4/Zn4	3.2180(19)	×4		Ag12	2.8637(16)	×4
	Ag12	3.165(3)	×4	Ag5	Ag5	2.860(8)	
	Ag12	3.1652(12)	×2		Zn5	2.82(4)	
	Zn15	3.020(2)	×6		Ag6	2.874(6)	
Y2	Ag2/Zn2	3.116(2)	×2		Ag6	2.874(4)	
	Ag5	3.158(6)	×2		Zn6	2.812(7)	
	Zn5	3.19(3)	×2		Zn6	2.812(6)	
	Ag6	3.176(2)			Ag13	2.875(5)	
	Ag6	3.176(3)	×3		Ag13	2.875(4)	
	Zn6	3.120(4)	×2		Zn11	2.601(5)	
	Zn6	3.120(5)			Zn14	2.769(6)	
	Zn6	3.120(6)			Zn14	2.769(4)	
	Ag11	3.028(3)		Zn5	Zn5	2.77(5)	
	Ag11	3.028(4)			Ag6	2.95(3)	
	Ag13	3.2546(15)	×2		Ag6	2.95(2)	
	Ag13	3.255(2)	×2		Ag13	2.86(3)	
	Zn12	3.151(3)			Ag13	2.856(19)	
Y3	Y3	3.640(3)			Zn11	2.69(3)	
	Ag1/Zn1	3.3384(19)			Zn14	2.68(3)	
	Ag2/Zn2	3.088(3)			Zn14	2.68(2)	
	Ag2/Zn2	3.089(3)		Ag6	Ag7	2.580(7)	
	Ag3/Zn3	3.2405(19)			Zn7	2.68(2)	×2
	Ag3/Zn3	3.240(3)			Ag13	2.789(2)	
	Ag4/Zn4	3.2535(19)			Ag13	2.789(3)	
	Ag8	3.265(3)			Zn11	2.746(2)	
	Ag10	3.20(5)			Zn12	2.622(2)	
	Ag10	3.20(3)		Zn6	Ag7	2.840(8)	
	Zn10	3.117(16)			Zn11	2.516(5)	
	Zn10	3.117(10)			Zn12	2.340(5)	
	Ag11	3.122(2)	×2	Ag7	Ag7	2.983(11)	
	Ag12	3.1441(12)			Zn7	2.89(3)	×2
	Ag12	3.144(3)			Zn8	2.588(7)	×2
	Ag13	3.105(3)			Ag13	2.911(4)	×4
	Ag13	3.1055(12)			Zn13	2.822(4)	×2
	Ag14	3.290(4)		Zn7	Zn7	2.82(4)	
	Ag14	3.290(3)			Zn7	2.77(4)	
	Zn14	3.168(2)			Zn8	2.76(3)	
	Zn14	3.1679(18)			Zn8	2.300(15)	
Y4	Ag1/Zn1	3.159(2)	×2		Ag8	2.801(13)	
	Ag5	3.192(6)	×2		Ag13	2.737(13)	×2

	Ag5	3.192(4)	×2
	Zn5	3.20(2)	×2
	Zn5	3.20(3)	×2
	Ag6	3.151(2)	×2
	Zn6	3.292(6)	×2
	Ag13	3.311(2)	×4
	Zn11	3.3134(12)	×2
	Zn14	3.238(3)	×2
Ag1	Ag13	3.0199(15)	×4
Ag1/Zn1	Zn13	2.7484(18)	×2
	Zn14	2.691(2)	×2
Ag2	Ag3/Zn3	2.7450(17)	
	Ag5	2.840(5)	
	Ag10	2.78(5)	
	Ag10	2.78(4)	
	Zn10	2.801(17)	
	Zn10	2.801(14)	
	Ag11	2.752(2)	×2
	Ag12	2.999(3)	
	Ag13	2.898(3)	
	Ag13	2.8982(12)	
	Zn14	2.7931(17)	
	Zn14	2.7931(15)	
Zn2	Ag3/Zn3	2.7450(17)	
	Ag5	2.840(5)	
	Zn5	2.77(3)	
	Ag10	2.78(5)	
	Ag10	2.78(4)	
	Zn10	2.801(17)	
	Zn10	2.801(14)	
	Ag11	2.752(2)	×2
	Ag13	2.898(3)	
	Ag13	2.8982(12)	
	Zn14	2.7931(17)	
	Zn14	2.7931(15)	
Ag3	Ag4/Zn4	2.640(2)	
	Ag4/Zn4	2.640(3)	
	Ag12	2.8234(17)	×2
	Zn14	2.697(3)	
	Zn14	2.697(2)	
Zn3	Ag4/Zn4	2.640(2)	
	Ag4/Zn4	2.640(3)	
	Ag12	2.8234(17)	×2
	Zn14	2.697(3)	
	Zn13	2.781(15)	×2
Zn8	Ag11	2.612(4)	×2
	Ag13	2.710(2)	×4
Ag8	Ag10	2.93(5)	×2
	Zn10	2.712(14)	×2
	Ag11	2.151(4)	×2
	Ag13	2.795(3)	×4
Ag9	Ag9	2.28(3)	
	Ag9	2.28(6)	
	Zn9	2.49(4)	
	Zn9	2.49(7)	
	Ag10	2.85(8)	
	Ag10	2.85(11)	
	Ag12	2.671(7)	×4
	Ag14	2.972(7)	×2
	Zn15	2.62(4)	×2
Zn9	Zn9	2.66(4)	
	Zn9	2.66(8)	
	Ag10	2.54(8)	
	Ag10	2.54(11)	
	Zn10	2.64(6)	×2
	Ag12	2.6400(18)	×2
	Ag12	2.6401(18)	×2
Ag10	Ag11	2.64(8)	
	Ag12	2.91(3)	×2
Zn10	Zn10	2.37(4)	
	Ag11	2.79(2)	
Ag11	Ag11	3.018(4)	
	Ag11	3.021(4)	
	Ag14	2.491(5)	
	Ag14	2.715(5)	
Ag12	Ag14	2.663(3)	
	Ag14	2.880(4)	
	Ag14	2.879(4)	
	Ag14	2.663(4)	
	Zn15	2.808(2)	
Ag13	Zn13	2.678(2)	
	Zn14	2.7775(17)	
Ag14	Zn15	2.450(4)	
	Zn15	2.549(5)	
Zn11	Zn11	2.566(3)	
Zn14	Zn14	2.789(2)	
Zn15	Zn15	2.564(4)	

S3. Wavelength Dispersive X-ray Spectroscopy (WDS) Results

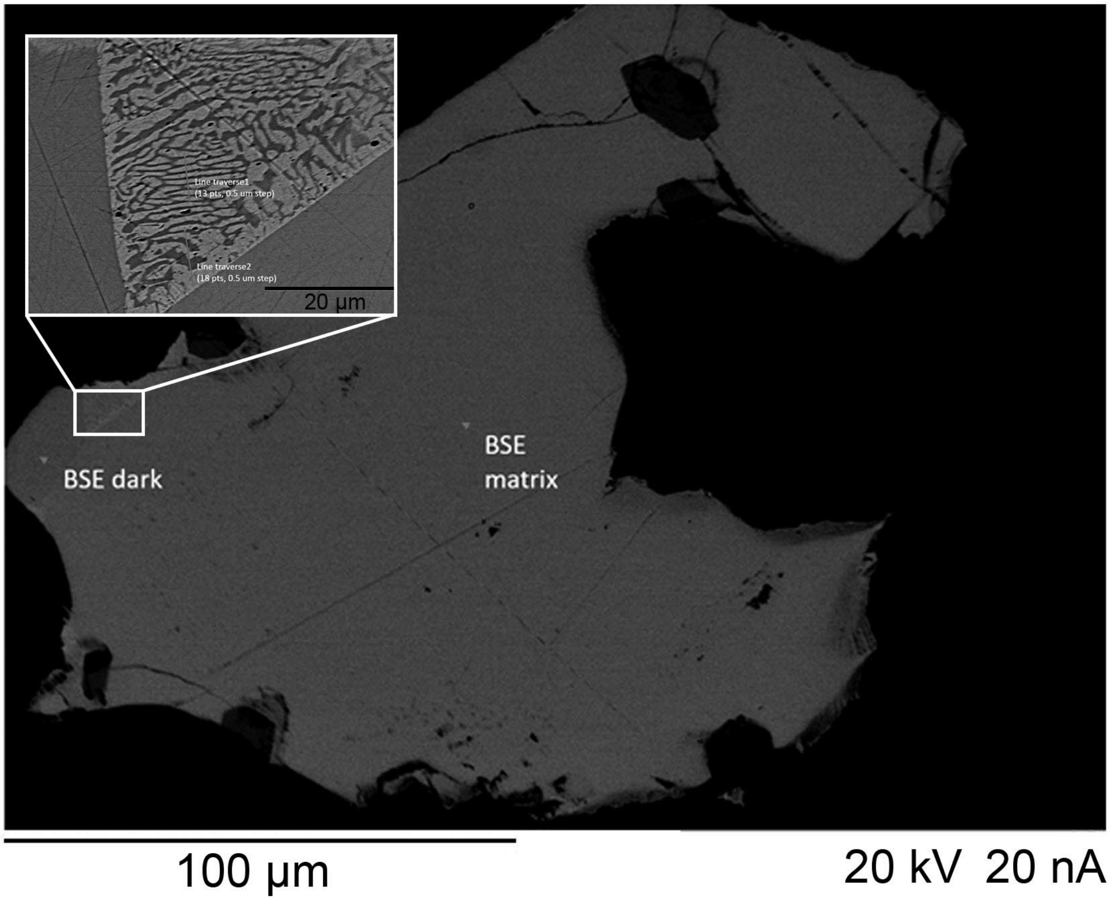

Figure S2. SEM-BSE image of a grain from a sample for which the powder X-ray diffraction pattern showed $Y_{13}Ag_{42.7}Zn_{29.7}$ to be the major phase. The inset shows a close up of a region containing a eutectic composition product.

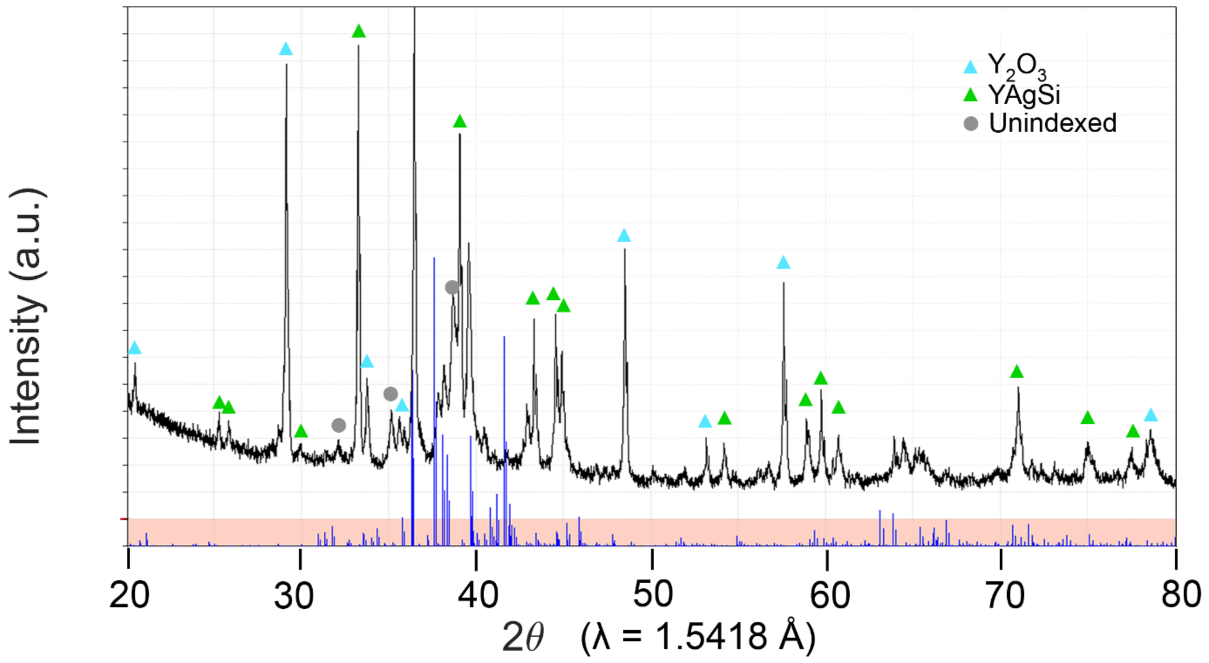

Figure S3. Powder X-ray diffraction pattern for the product of the stoichiometric synthesis of $Y_{13}Ag_{42.7}Zn_{29.7}$. Calculated intensities for $Y_{13}Ag_{42.7}Zn_{29.7}$ are given for comparison, while peaks attributed to YAgSi, Y_2O_3, or an unknown impurity are indicated.

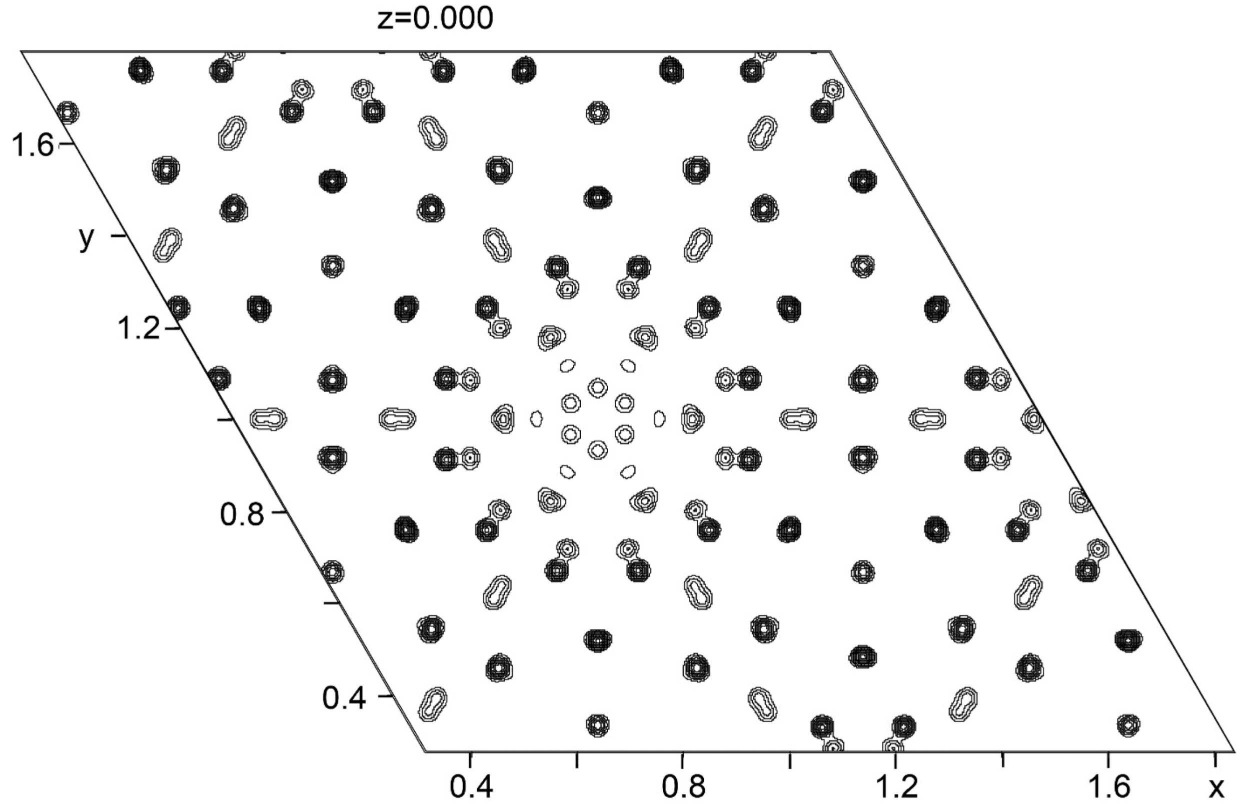

Figure S4. Fourier electron density contours for the z=0.0 layer of $Y_{13}Ag_{42.7}Zn_{29.7}$ derived from single crystal X-ray diffraction data collected at T = 100 K. Comparison with Figures 3 and 6 show that the electron density features associated with the Ag9 and Zn7/Ag7 sites have grown less elongated along the radial direction from the high-symmetry (x, y)=$(0, 0)$ point. This element of the disorder thus has a relatively strong vibrational component.

Table S4. WDS compositions measurements for different points corresponding to the $Y_{13}Ag_{42.7}Zn_{29.7}$ matrix phase.

SAMPLE	LINE	**Ag AT%**	**Y AT%**	**Zn AT%**	**TOTAL**
sam2img1 BSE-matrix	236	49.73709	15.23435	35.02847	100
sam2img1 BSE-matrix	237	50.03819	15.15665	34.80519	100
sam2img1 BSE-matrix	238	50.03993	15.08845	34.87162	100
sam2img1 BSE-matrix	239	50.37801	15.10206	34.51990	100
sam2img1 BSE-matrix	240	49.92185	15.19702	34.88118	100
sam2img1 BSE-matrix	241	50.29345	15.00724	34.69937	100
sam2img1 BSE-matrix	242	50.83458	14.77427	34.39108	100
sam2img1 BSE-matrix	243	49.79255	15.35778	34.84973	100
sam2img1 BSE-matrix	244	50.13727	15.17569	34.68707	100
sam2img1 BSE-matrix	245	49.84505	15.05418	35.10071	100
sam2img1 BSE-matrix	246	50.14915	15.22177	34.62912	100
sam2img1 BSE-matrix	247	50.18903	14.99081	34.82010	100
sam2img1 BSE-matrix	248	49.97185	15.22809	34.80009	100
sam2img1 BSE-matrix	249	51.02227	14.65534	34.32246	100
sam2img1 BSE-matrix	250	50.28496	15.09329	34.62175	100
sam2img1 BSE-matrix	251	49.97952	15.10707	34.91347	100
sam2img1 BSE-matrix	252	50.19088	14.98310	34.82600	100
sam2img1 BSE-matrix	253	50.19559	15.20426	34.60015	100
sam2img1 BSE-matrix	254	49.97171	15.08204	34.94632	100
sam2img1 BSE-matrix	255	49.97464	15.20476	34.82071	100
sam2img2 BSE-matrix	276	49.88415	15.07513	35.04076	100
sam2img2 BSE-matrix	277	50.18102	15.13590	34.68309	100
sam2img2 BSE-matrix	278	49.98588	15.31299	34.70110	100
sam2img2 BSE-matrix	279	49.88085	15.29611	34.82303	100
sam2img2 BSE-matrix	280	49.96149	15.00382	35.03464	100
sam2img2 BSE-matrix	281	50.11930	15.23131	34.64944	100
sam2img2 BSE-matrix	282	49.80534	15.03809	35.15647	100
sam2img2 BSE-matrix	283	49.93116	15.34216	34.72662	100
sam2img2 BSE-matrix	284	50.04276	15.09943	34.85788	100
sam2img2 BSE-matrix	285	50.07250	15.00351	34.92399	100
sam2img2 BSE-matrix	286	50.27530	15.26290	34.46179	100
sam2img2 BSE-matrix	287	50.52500	15.01581	34.45922	100
sam2img2 BSE-matrix	288	49.84585	15.10205	35.05214	100
sam2img2 BSE-matrix	289	50.11138	15.15018	34.73853	100
sam2img2 BSE-matrix	290	49.95515	15.05245	34.99236	100
sam2img2 BSE-matrix	291	49.80328	15.27080	34.92590	100
sam2img2 BSE-matrix	292	50.06620	15.12300	34.81073	100
sam2img2 BSE-matrix	293	50.26059	14.74968	34.98967	100
sam2img2 BSE-matrix	294	50.10300	14.95301	34.94408	100
sam2img2 BSE-matrix	295	49.97435	15.07538	34.95031	100
Average of 40 points		50.09330	15.10530	34.80141	
Standard deviation		0.256949	0.149377	0.195646	

Table S5. WDS composition measurements for different points corresponding to the dark phase in the $Y_{13}Ag_{42.7}Zn_{29.7}$ sample.

SAMPLE	LINE	Ag AT%	Y AT%	Zn AT%	TOTAL
sam2img1 BSE-dark	256	47.75036	15.76433	36.48543	100
sam2img1 BSE-dark	257	47.86908	15.66672	36.46418	100
sam2img1 BSE-dark	258	47.81020	15.86000	36.32978	100
sam2img1 BSE-dark	259	48.04005	15.70399	36.25585	100
sam2img1 BSE-dark	260	48.45405	15.42174	36.12413	100
sam2img1 BSE-dark	261	48.41897	15.46760	36.11346	100
sam2img1 BSE-dark	262	48.20938	15.45012	36.34046	100
sam2img1 BSE-dark	263	47.90941	15.58716	36.50352	100
sam2img1 BSE-dark	264	48.15943	15.68618	36.15439	100
sam2img1 BSE-dark	265	48.31421	15.55959	36.12627	100
sam2img1 BSE-dark	266	48.31574	15.75108	35.93324	100
sam2img1 BSE-dark	267	48.15038	15.72028	36.12932	100
sam2img1 BSE-dark	268	48.19307	15.44841	36.35854	100
sam2img1 BSE-dark	269	48.38086	15.49032	36.12887	100
sam2img1 BSE-dark	270	48.05331	15.76556	36.18106	100
sam2img1 BSE-dark	271	48.13656	15.38713	36.47633	100
sam2img1 BSE-dark	272	48.16311	15.58732	36.24958	100
sam2img1 BSE-dark	273	47.89040	15.76903	36.34066	100
sam2img1 BSE-dark	274	48.24604	15.43579	36.31823	100
sam2img1 BSE-dark	275	48.27509	15.45979	36.26517	100
sam2img2 BSE-dark	296	47.77997	15.69880	36.52126	100
sam2img2 BSE-dark	297	47.96852	15.78357	36.24797	100
sam2img2 BSE-dark	298	47.73132	16.11450	36.15418	100
sam2img2 BSE-dark	299	47.81720	15.70747	36.47539	100
sam2img2 BSE-dark	300	47.99407	15.77844	36.22751	100
sam2img2 BSE-dark	301	48.15449	15.75202	36.09345	100
sam2img2 BSE-dark	302	47.76667	15.60678	36.62661	100
sam2img2 BSE-dark	303	48.04350	15.84649	36.11012	100
sam2img2 BSE-dark	304	48.08999	15.72761	36.18241	100
sam2img2 BSE-dark	305	48.05551	15.53969	36.40482	100
sam2img2 BSE-dark	306	48.37364	15.52373	36.10263	100
sam2img2 BSE-dark	307	48.03241	16.06320	35.90440	100
sam2img2 BSE-dark	308	48.26580	15.56636	36.16783	100
sam2img2 BSE-dark	309	48.00300	15.70636	36.29065	100
sam2img2 BSE-dark	310	47.93513	15.65729	36.40757	100
sam2img2 BSE-dark	311	47.73590	15.96664	36.29751	100
sam2img2 BSE-dark	312	47.85772	15.97736	36.16495	100
sam2img2 BSE-dark	313	47.86660	15.75615	36.37718	100
sam2img2 BSE-dark	314	47.81145	15.97484	36.21370	100
sam2img2 BSE-dark	315	47.95794	15.92482	36.11729	100
Average of 40 points		48.04951	15.69136	36.25915	
Standard deviation		0.20524	0.181392	0.159192	

S4. Analysis of Mg_2Zn_{11}-Type Domains within the $Y_{13}Ag_{42.7}Zn_{29.7}$ Structure

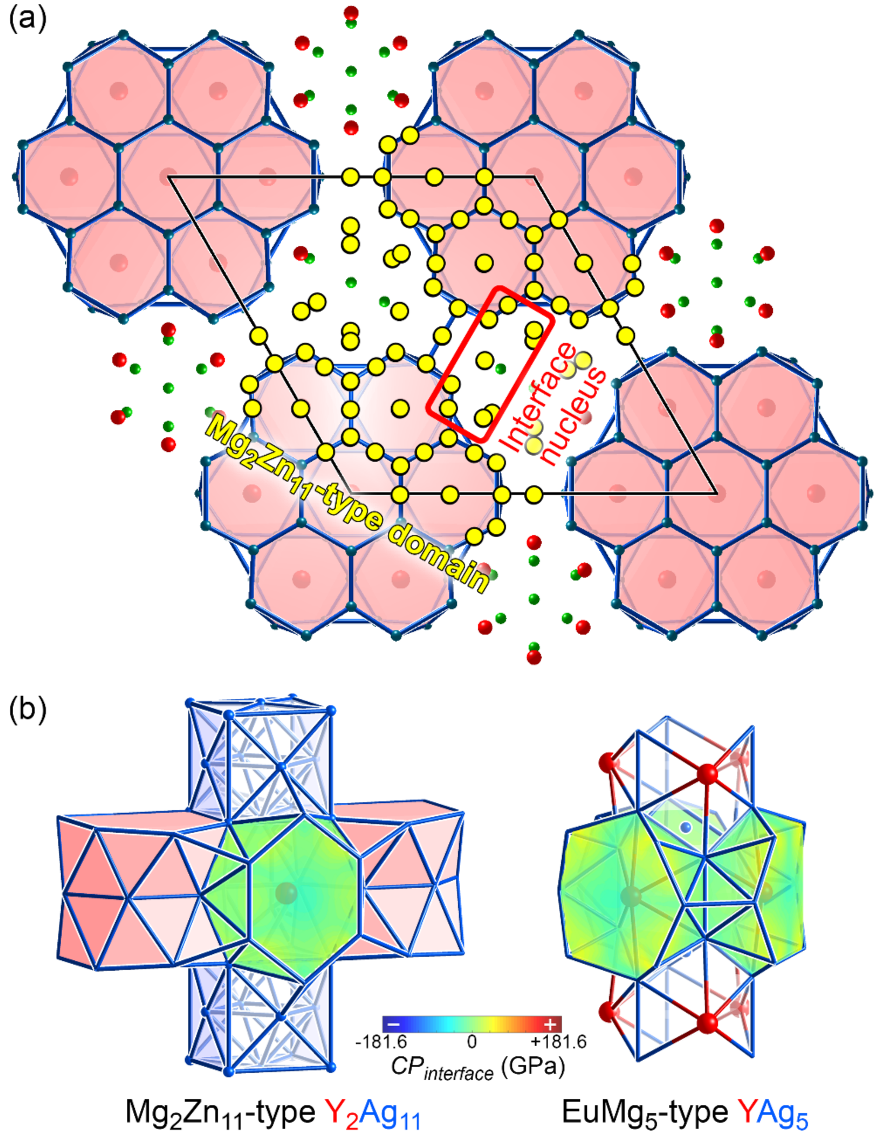

Figure S5. Role of the Mg_2Zn_{11} parent structure in $Y_{13}Ag_{42.7}Zn_{29.7}$. (a) The ordered portion of the $Y_{13}Ag_{42.7}Zn_{29.7}$ structure overlaid with atomic positions from the Mg_2Zn_{11} structure type (yellow) mapped to it by the *GrowDomain* program. The Mg_2Zn_{11}-type domain bridges two $CaPd_{5+x}$-based discs, encompassing almost half of each, and spreads into the $EuMg_{5+x}$ domains. The strong CP complementarity between the Mg_2Zn_{11} and $CaPd_{5+x}$ parent phases was evaluated earlier in Fredrickson, R. T.; Fredrickson, D. C. *Inorg. Chem.* **2024**, *63*, 9252-9264. (b) $CP_{interface}$ functions for a potential interface nucleus between the Mg_2Zn_{11}-type and $EuMg_5$-type domains, a distorted hexagonal antiprism that is shared between them in $Y_{13}Ag_{42.7}Zn_{29.7}$. The $CP_{interface}$ functions are calculated for Y-Ag versions of the structures. Little complementarity is seen between the functions between these two parent structures. The driving forces for the formation of the structure are then attributed to the $CaPd_{5+x}$-type/Mg_2Zn_{11}-type and $CaPd_{5+x}$-type/$EuMg_5$-type interfaces, with the shared motif at the Mg_2Zn_{11}-type/$EuMg_5$-type interface supporting the geometrical feasibility of the modular arrangement.

S5. Additional Computational Details

Table S6. Unit cell vectors for the LDA-DFT-optimized structures in Cartesian Coordinates.

Formula	Structure type/total E	Vector	*x* (Å)	*y* (Å)	*z* (Å)
YAg_5	$EuMg_5$	**a**	9.20167	0	0
	-846.19808 eV/atom	**b**	-4.60083	7.96888	0
		**c**	0	0	8.93248
YAg_5	$CaPd_{5+x}$ (simplified)	**a**	5.24849	0	0
	-846.25827 eV/atom	**b**	-2.62425	4.54533	0
		**c**	0	0	9.15824

Table S7. LDA-DFT-optimized fractional atomic coordinates for $EuMg_5$-type YAg_5.

Element	*x*	*y*	*z*
Y	0.18409	0.36818	0.25000
Ag	0.00000	0.00000	0.00000
Ag	0.33333	0.66667	0.03422
Ag	0.50000	0.00000	0.00000
Ag	0.56762	0.13524	0.25000
Ag	0.83910	0.67820	0.08858
Y	0.81591	0.18409	0.75000
Ag	0.00000	0.00000	0.50000
Ag	0.66667	0.33333	0.53422
Ag	0.50000	0.50000	0.50000
Ag	0.43238	0.56762	0.75000
Ag	0.16090	0.83910	0.58858
Y	0.36818	0.18409	0.75000
Ag	0.00000	0.50000	0.50000
Ag	0.13524	0.56762	0.75000
Ag	0.67820	0.83910	0.58858
Y	0.63182	0.81591	0.25000
Ag	0.00000	0.50000	0.00000
Ag	0.86476	0.43238	0.25000
Ag	0.32180	0.16090	0.08858
Y	0.18409	0.81591	0.25000
Ag	0.50000	0.50000	0.00000
Ag	0.56762	0.43238	0.25000
Ag	0.83910	0.16090	0.08858
Y	0.81591	0.63182	0.75000
Ag	0.50000	0.00000	0.50000
Ag	0.43238	0.86476	0.75000
Ag	0.16090	0.32180	0.58858
Ag	0.66667	0.33333	0.96578

Ag	0.16090	0.32180	0.91142
Ag	0.33333	0.66667	0.46578
Ag	0.83910	0.16090	0.41142
Ag	0.32180	0.16090	0.41142
Ag	0.67820	0.83910	0.91142
Ag	0.16090	0.83910	0.91142
Ag	0.83910	0.67820	0.41142

Table S8. LDA-DFT-optimized fractional atomic coordinates for $CaPd_{5+x}$ (simplified)-type YAg_5.

Element	*x*	*y*	*z*
Y	0.66667	0.33333	0.28971
Y	0.66667	0.33333	0.71029
Ag	0.18761	0.33332	0.50000
Ag	0.14571	0.81239	0.50000
Ag	0.66668	0.85429	0.50000
Ag	0.33333	0.66667	0.76357
Ag	0.33333	0.66667	0.23643
Ag	0.00000	0.00000	0.23643
Ag	0.00000	0.00000	0.76357
Ag	0.34545	0.01207	0.00000
Ag	-0.01207	0.33338	0.00000
Ag	0.66662	0.65455	0.00000
